# A circadian output center controlling feeding:fasting rhythms in *Drosophila*

**DOI:** 10.1371/journal.pgen.1008478

**Published:** 2019-11-06

**Authors:** Austin P. Dreyer, Madison M. Martin, Carson V. Fulgham, Daniel A. Jabr, Lei Bai, Jennifer Beshel, Daniel J. Cavanaugh

**Affiliations:** 1 Department of Biology, Loyola University Chicago, Chicago, Illinois, United States of America; 2 Penn Chronobiology, Howard Hughes Medical Institute, Perelman School of Medicine, University of Pennsylvania, Philadelphia, Pennsylvania, United States of America; Universidad de Valparaiso, CHILE

## Abstract

Circadian rhythms allow animals to coordinate behavioral and physiological processes with respect to one another and to synchronize these processes to external environmental cycles. In most animals, circadian rhythms are produced by core clock neurons in the brain that generate and transmit time-of-day signals to downstream tissues, driving overt rhythms. The neuronal pathways controlling clock outputs, however, are not well understood. Furthermore, it is unclear how the central clock modulates multiple distinct circadian outputs. Identifying the cellular components and neuronal circuitry underlying circadian regulation is increasingly recognized as a critical step in the effort to address health pathologies linked to circadian disruption, including heart disease and metabolic disorders. Here, building on the conserved components of circadian and metabolic systems in mammals and *Drosophila melanogaster*, we used a recently developed feeding monitor to characterize the contribution to circadian feeding rhythms of two key neuronal populations in the *Drosophila* pars intercerebralis (PI), which is functionally homologous to the mammalian hypothalamus. We demonstrate that thermogenetic manipulations of PI neurons expressing the neuropeptide SIFamide (SIFa) as well as mutations of the *SIFa* gene degrade feeding:fasting rhythms. In contrast, manipulations of a nearby population of PI neurons that express the *Drosophila* insulin-like peptides (DILPs) affect total food consumption but leave feeding rhythms intact. The distinct contribution of these two PI cell populations to feeding is accompanied by vastly different neuronal connectivity as determined by trans-Tango synaptic mapping. These results for the first time identify a non-clock cell neuronal population in *Drosophila* that regulates feeding rhythms and furthermore demonstrate dissociable control of circadian and homeostatic aspects of feeding regulation by molecularly-defined neurons in a putative circadian output hub.

## Author summary

Circadian (~24-hr) rhythms allow organisms to organize behavioral and physiological processes with respect to one another and the external environment. Circadian information is generated by central clock neurons in the brain that keep time through the presence of molecular clocks. To modulate behavioral processes, circadian signals must be transmitted through output pathways to control relevant downstream neuronal populations. We have investigated control of feeding behavior by two molecularly-distinct populations of neurons in a putative circadian output center in the fruit fly, *Drosophila melanogaster*. We identify for the first time a population of neurons, marked by expression of the SIFa peptide, that act as part of the circadian output circuit controlling feeding:fasting rhythms, and furthermore show that SIFa expression within these cells is necessary for normal feeding rhythms. Interestingly, manipulation of a nearby population of neurons that express the *Drosophila* insulin-like peptides alters the amount of feeding independent of effects on feeding rhythms, indicating that circadian and homeostatic aspects of feeding behavior are regulated by independent neuronal subsets. These findings have important implications for our understanding of how the central clock coordinately modulates distinct behavioral outputs.

## Introduction

Most physiological and behavioral processes exhibit ~24 hr rhythms that are maintained by a network of circadian clock cells and synchronized to environmental conditions. The capacity to anticipate and respond appropriately to daily environmental cycles is a crucial factor in organismal fitness, as evidenced by the presence of circadian regulatory systems across taxa [1]. The importance of circadian rhythms is further demonstrated by the negative effects associated with their disruption, which include reduced lifespan and metabolic disorders, among other health pathologies, in invertebrates and mammals [2,3]. The correlation between circadian disruption and metabolic disorders extends to humans as well, with an emphasis on the importance of timing of feeding on metabolic health [4]. A deeper understanding of how the circadian system regulates behavioral outputs such as rest:activity patterns or feeding:fasting rhythms, and ensures their appropriate timing with physiological processes such as growth and metabolism, is necessary to address the far-reaching impacts of circadian disruption.

Our understanding of circadian systems has greatly benefited from research using the fruit fly, *Drosophila melanogaster*, including the discovery of core clock genes and delineation of the molecular mechanisms through which cells keep time [5,6]. Importantly, these mechanisms are highly conserved between fruit flies and mammals. Research in the past ~50 years has shown that circadian rhythms are dictated by a set of core clock, or pacemaker, neurons in the brain. In *Drosophila*, ~150 neurons distributed throughout the brain comprise the core clock, which is analogous to the suprachiasmatic nucleus in the hypothalamus of mammals. These cells track time of day through a molecular clock that operates as a transcriptional-translational feedback loop and establishes 24-hr oscillations of gene expression [6–9].

In addition to core clock neurons, circadian systems are made up of input pathways, which synchronize the internal clock to environmental conditions by transmitting information about environmental stimuli (e.g. light or temperature), and output pathways, which conduct circadian signals to the appropriate tissues via cellular and molecular signals [6,9,10]. Input pathways and the core clock have been the focus of circadian research for decades, but the structure of output pathways has only recently begun to be elucidated. Furthermore, most circadian rhythm research in flies has focused on the regulation of rest:activity rhythms; however, an emerging question is how a single central clock is able to regulate multiple behavioral outputs which may have unique temporal patterns and rely on distinct downstream output circuitry.

The *Drosophila* pars intercerebralis (PI), a functional homolog of the mammalian hypothalamus [11] has recently been identified as a circadian output center in the fly [12]. The PI is the source of multiple signaling molecules, including diuretic hormone 44 (DH44), SIFamide (SIFa) and the *Drosophila* insulin-like peptides (DILPs), which define non-overlapping PI subsets [13] that appear to differentially contribute to circadian outputs. Thus, genetic manipulations that ablate or constitutively activate DH44+ cells, or that deplete DH44 peptide, degrade rest:activity rhythm strength, whereas manipulations of the DILP+ neurons, which are collectively known as the insulin-producing cells (IPCs), have no effect [12]. The contribution of SIF+ neurons to rest:activity rhythms is less clear. Ablation of these cells reduces locomotor rhythmicity, but mutant flies lacking SIFa peptide were found to have inconsistent circadian phenotypes [14].

Interestingly, although DH44 signaling is essential for the production of normal rest:activity rhythms, it is dispensable for rhythms of feeding [15], demonstrating separable control of these two behaviors and suggesting that circadian regulation of feeding and locomotor activity may diverge at the level of the PI output cell. We therefore reasoned that non-DH44-expressing PI neurons could comprise part of the circadian output pathway controlling feeding behavior. This is supported by the fact that, like DH44+ cells, the DILP+ and SIFa+ subsets are functionally and/or anatomically connected to core clock cells, and the IPCs additionally exhibit circadian patterns of neuronal activity [12,16,17]. Furthermore, SIFa+ and DILP+ PI cells have been ascribed functions relating to feeding and/or metabolism, although not yet to the circadian regulation of those processes. The IPCs have demonstrated roles in the regulation of metabolism, tissue growth and lifespan [18].They have also been implicated in regulating feeding behavior, for example by suppressing foraging and food intake under conditions of satiety in *Drosophila* larvae, although such a role has not always been found [19,20]. SIFamide was originally characterized for its role in the modulation of courtship behavior and sleep [14,21,22], but recently was also found to affect appetitive behavior and food intake by altering olfactory responsiveness to food cues during starvation [23].

Here, we tested the impact of DILP+ and SIFa+ cells on circadian feeding regulation using the recently-developed fly liquid-food interaction counter (FLIC) system that allows for real-time recording of *Drosophila* feeding behavior over the timescale necessary for circadian analysis [24]. Importantly, the FLIC system has confirmed feeding:fasting rhythms are under control of the central clock, as *period* mutant flies exhibit arrhythmic feeding in DD conditions [24]. Using restricted GAL4 drivers, we acutely activated or silenced each subset of PI cells while continuously recording feeding behaviors. We find that adult-specific activation of SIFa+ cells and mutations that eliminate SIFa expression disrupt feeding rhythms. In contrast, activation of DILP+ PI cells leaves feeding rhythms intact but increases overall food consumption. Our results indicate dissociable control of circadian and homeostatic aspects of feeding by these distinct PI neuron subsets. Experiments using the trans-Tango technique [25] to identify postsynaptic targets of DILP+ and SIFa+ PI cells further provide a potential anatomical basis for the differential contribution to feeding of the two cell populations. In comparison to the relatively restricted synaptic connectivity of the DILP+ cells, SIFa+ neurons make extensive synaptic connections throughout the brain, including to areas involved in odor detection and feeding, as well as onto core clock cells. The distinct connectivity patterns could indicate that IPCs signal systemically to transmit information relating to overall food intake, while SIFa+ cells integrate circadian information and act synaptically to modulate the timing of feeding.

## Results

### SIFa+ PI cells regulate circadian feeding:fasting rhythms

**Fig 1:**
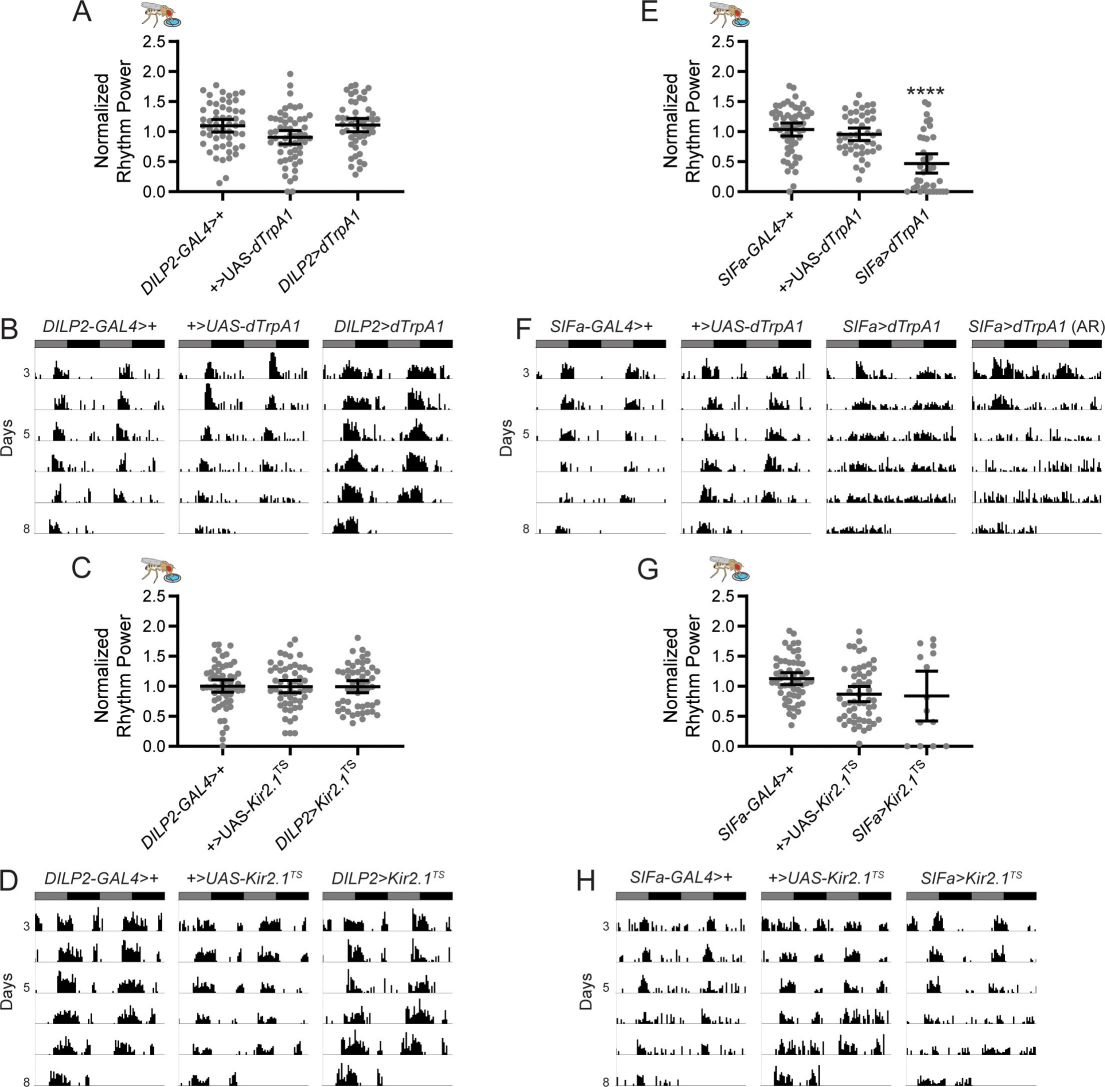
Activation of SIFa+ cells weakens feeding:fasting rhythms. (A) Adult-specific, *dTrpA1*-mediated activation of the IPCs has no effect on feeding rhythms as compared to genetic controls. Normalized feeding rhythm power is plotted for the indicated genotypes. Dots represent strength of individual fly feeding rhythms and lines represent mean ± 95% confidence interval. (B) Representative single-fly feeding records are shown for experimental days 3–8 for the indicated genotypes. Flies were transferred to DD conditions and exposed to elevated temperatures at the start of experimental day 2. Feeding records show number of feeding events in 30 min bins, and data are double plotted, with each line representing two days of data. Gray and black bars represent subjective day and night, respectively. (C-D) Adult-specific, *Kir2*.*1*-mediated silencing of IPCs also has no effect on feeding rhythm strength. (C) Normalized feeding rhythm power is plotted for the indicated genotypes as described for (A). (D) Representative single-fly feeding records are shown for the indicated genotypes as described for (B). (E-F) Adult-specific, *dTrpA1*-mediated activation of SIFa+ cells significantly reduces feeding rhythm strength. (E) Normalized feeding rhythm power is plotted for the indicated genotypes as described in (A). (F) Representative single-fly feeding records are shown for the indicated genotypes as described for (B). For *SIFa*>*dTrpA1* flies, two records are shown: one for a fly that exhibited a rhythm power consistent with the group mean, and another for one of the ~35% of *SIFa*>*dTrpA1* flies that were arrhythmic (AR). (G-H) Adult-specific, *Kir2*.*1*-mediated silencing of SIFa+ cells in adult flies results in high lethality, with no effect on mean feeding rhythm strength in the surviving *SIFa*>*Kir2*.*1*^TS^ flies. (G) Normalized feeding rhythm power is plotted for the indicated genotypes as described in (A). (H) Representative single-fly feeding records are shown for the indicated genotypes as described for (B). For rhythm power plots, ****p<0.0001, Tukey’s multiple comparisons test for experimental cross compared to both control lines. See S1 Table for exact n and *p*-value for each experiment.

**Fig 2:**
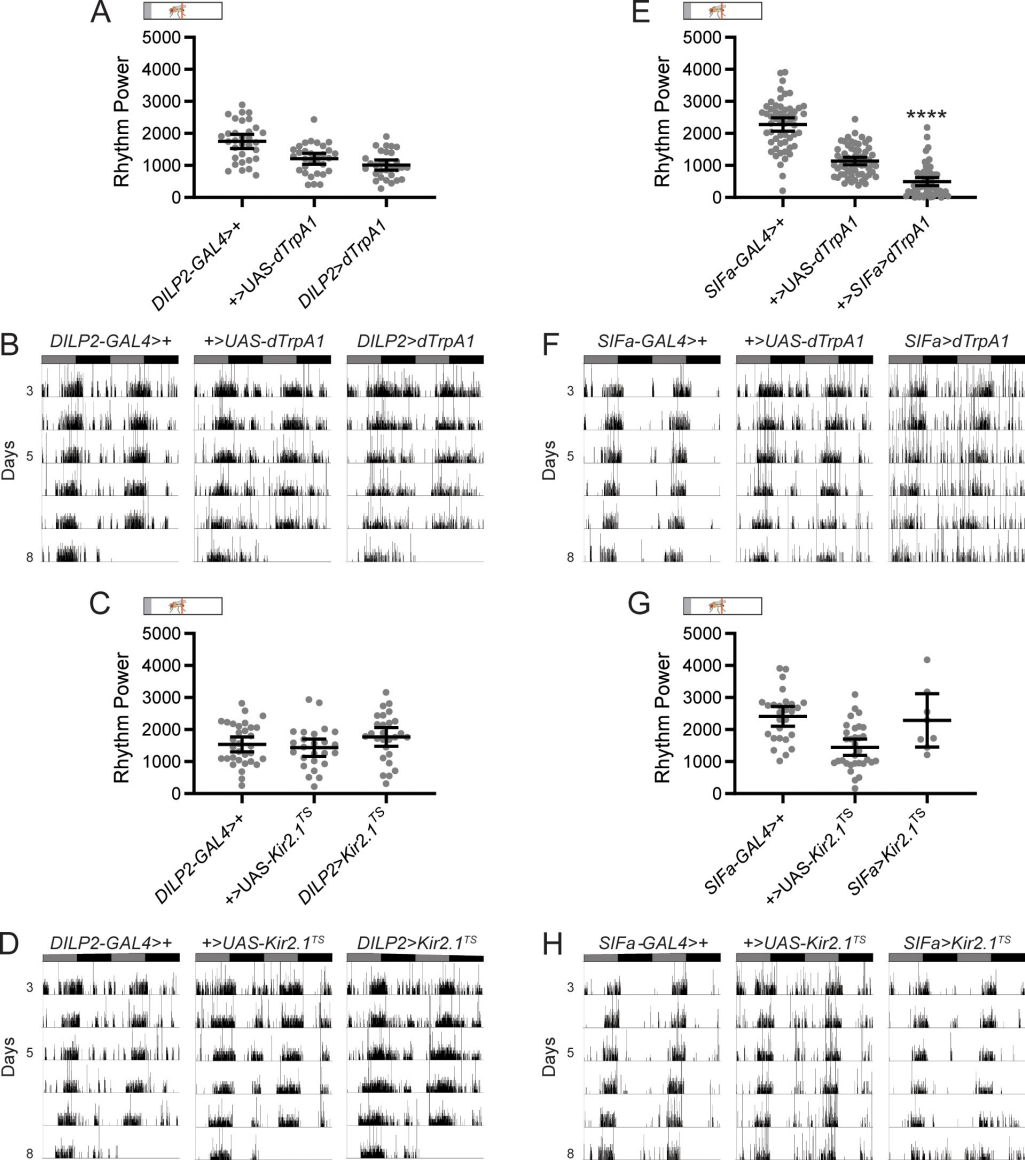
Activation of SIFa+ cells weakens rest:activity rhythms. (A) Adult-specific, *dTrpA1*-mediated activation of the IPCs has no effect on locomotor rhythms as compared to genetic controls. Rest:activity rhythm power is plotted for the indicated genotypes. Dots represent strength of individual fly rest:activity rhythms and lines represent mean ± 95% confidence interval. (B) Representative single-fly activity records are shown for experimental days 3–8 for the indicated genotypes. Flies were transferred to DD conditions and exposed to elevated temperatures at the start of experimental day 2. Activity records show number of DAM beam breaks in 1 min bins, and data are double plotted, with each line representing two days of data. Gray and black bars represent subjective day and night, respectively. (C-D) Adult-specific, *Kir2*.*1*-mediated silencing of IPCs also has no effect on rest:activity rhythm strength. (C) Rest:activity rhythm power is plotted for the indicated genotypes as described for (A). (D) Representative single-fly activity records are shown for the indicated genotypes as described for (B). (E-F) Adult-specific, *dTrpA1*-mediated activation of SIFa+ cells significantly reduces rest:activity rhythm strength. (E) Rest:activity rhythm power is plotted for the indicated genotypes as described for (A). (F) Representative single-fly activity records are shown for the indicated genotypes as described for (B). (G-H) Adult-specific, *Kir2*.*1*-mediated silencing of SIFa+ cells results in high lethality, with no effect on mean rest:activity rhythm strength in the surviving *SIFa*>*Kir2*.*1*^TS^ flies. (G) Rest:activity rhythm power is plotted for the indicated genotypes as described for (A). (H) Representative single-fly activity records are shown for the indicated genotypes as described for (B). For rhythm power plots, ****p<0.0001, Tukey’s multiple comparisons test for experimental cross compared to both control lines. See S2 Table for exact n and *p*-values.

To test the contribution of PI neurons to feeding rhythms, we activated and silenced target cells using GAL4-driven expression of ion channels. We first targeted the IPCs using *DILP2*-*GAL4*, which is selectively expressed in these cells [26]. A potential circadian output function for IPCs is supported by the fact that they are physically and functionally connected to neurons of the core clock, and display circadian firing activity, with increased firing frequency in the morning as compared to the evening [17]. Nevertheless, manipulations of IPCs failed to alter feeding rhythms. Constitutive thermogenetic activation of DILP+ neurons in adult flies using the temperature-activated cation channel, *dTrpA1 [27],* had no effect on the period or strength of feeding:fasting rhythms (Fig 1A and 1B; S1 Table). Furthermore, normal feeding:fasting rhythms were observed in adult flies in which the IPCs were acutely silenced via GAL4-mediated expression of the inwardly rectifying potassium channel, *Kir2.1 [28]*, in combination with the TARGET system [29] to temporally control *Kir2*.*1* expression (Fig 1C and 1D; S1 Table). In agreement with previous results [12], we also found that rest:activity rhythms were unaffected following constitutive activation or silencing of IPCs (Fig 2A–2D; S2 Table). Taken together, there is no evidence for IPCs having a regulatory effect on circadian rhythms for feeding or locomotor behaviors.

As the DILP2+ PI cells did not appear to regulate feeding rhythms, we next tested for a potential contribution of SIFa+ neurons. We manipulated SIFa cells using a highly selective *SIFa*-*GAL4* [21]. In contrast to the IPC manipulations, feeding:fasting rhythms were disrupted following constitutive activation of adult SIFa+ neurons. Compared to genetic controls, a smaller percentage of *SIFa*>*dTrpA1* flies exhibited rhythmic feeding:fasting behavior (*p*<0.0001; Fisher’s exact test), and mean feeding:fasting rhythm strength was significantly reduced (Fig 1E and 1F; S1 Table). Feeding:fasting rhythm period length was unaltered, however, arguing against a direct effect on core clock mechanisms (S1 Table). Rhythm strength was unaffected compared to control lines in *SIFa*>*dTrpA1* flies maintained at 21°C, a temperature that prevents *dTrpA1*-mediated activation, demonstrating that the reduced rhythm strength of *SIFa*>*dTrpA1* flies is a specific effect of cell activation (S1 Fig).

**Fig 3:**
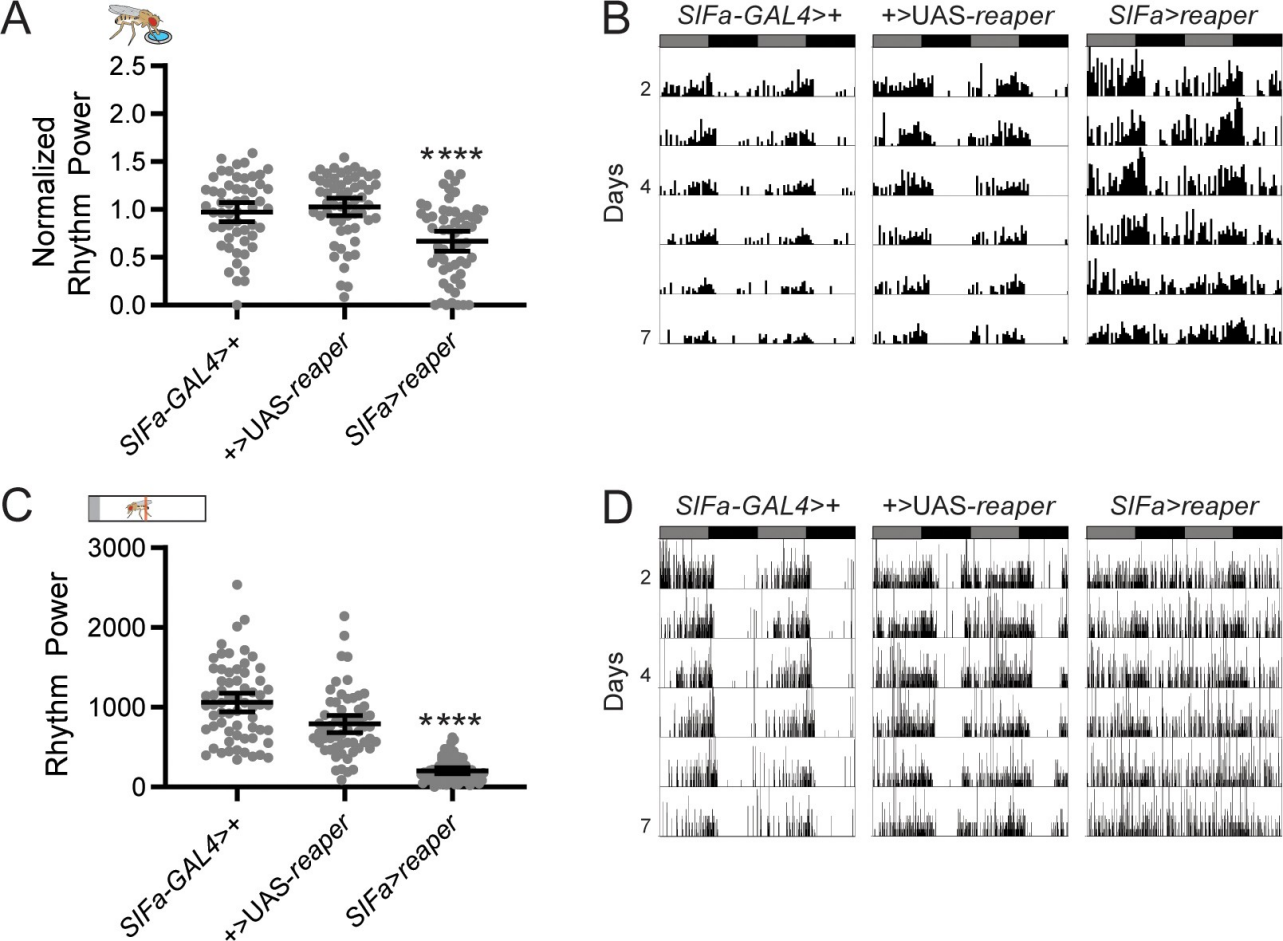
Ablation of SIFa+ cells weakens feeding:fasting and rest:activity rhythms. (A) Ablation of SIFa+ cells significantly reduces feeding rhythm strength. Normalized feeding rhythm power is plotted for the indicated genotypes. (B) Representative single-fly feeding records are shown for experimental days 2–7 for the indicated genotypes. Flies were transferred to DD conditions at the start of experimental day 2. Feeding records show number of feeding events in 30 min bins, and data are double plotted, with each line representing two days of data. Gray and black bars represent subjective day and night, respectively. (C) Ablation of SIFa+ cells significantly reduces locomotor rhythm strength. Rest:activity rhythm power is plotted for the indicated genotypes. (D) Representative single-fly activity records are shown for experimental days 2–7 for the indicated genotypes. Flies were transferred to DD conditions at the start of experimental day 2. Activity records show number of DAM beam breaks in 1 min bins, and data are double plotted, with each line representing two days of data. For rhythm power plots, dots represent strength of individual fly normalized feeding or rest:activity rhythms and lines represent mean ± 95% confidence interval, ****p<0.0001, Tukey’s multiple comparisons test for experimental cross compared to both control lines. See S1 and S2 Tables for exact n and *p*-values of feeding and locomotor results, respectively.

Surprisingly, we found that acute inhibition of SIFa+ PI cells resulted in high lethality, as 72.5% of flies exposed to adult-specific *Kir2*.*1*-mediated silencing died within a few hours to days of exposure to high temperatures. This precluded a thorough assessment of the consequences of SIFa+ cell silencing on feeding:fasting rhythm strength. Of the minority of flies that survived through the entire experiment, we found that many retained strong feeding:fasting rhythms, although we did note a higher percentage of arrhythmic flies in this group compared to controls (*p*<0.01; Fisher’s exact test). It is possible that some flies were spared the full effect of neuronal silencing, allowing them to survive the manipulation but also leaving feeding:fasting rhythms intact. To circumvent the lethality and still investigate the effect of manipulations that prevent SIFa signaling, we genetically ablated the SIFa+ cells by expressing the apoptosis-inducing transgene *reaper* [30]. SIFa+ cell ablated flies exhibited disrupted feeding:fasting rhythms with significantly reduced rhythm power as compared to genetic controls (Fig 3A and 3B; S1 Table).

We also tested how manipulation of SIFa+ cells affected rest:activity rhythms. Although we previously found no effect of constitutive activation of SIFa+ cells on rest:activity rhythm strength [12], here we found it to be significantly reduced in *SIFa*>*dTrpA1* flies compared to controls (Fig 2E and 2F; S2 Table). As with our feeding analysis, adult-specific silencing of SIFa+ cells resulted in high levels of lethality, with the few surviving flies showing normal rest:activity rhythm strength (Fig 2G and 2H; S2 Table). *SIFa*>*reaper* flies, however, exhibited significantly reduced rest:activity rhythm strength (Fig 3C and 3D, S2 Table), confirming an important contribution of these cells [12].

### SIFa Peptide is necessary for normal feeding:fasting rhythms

**Fig 4:**
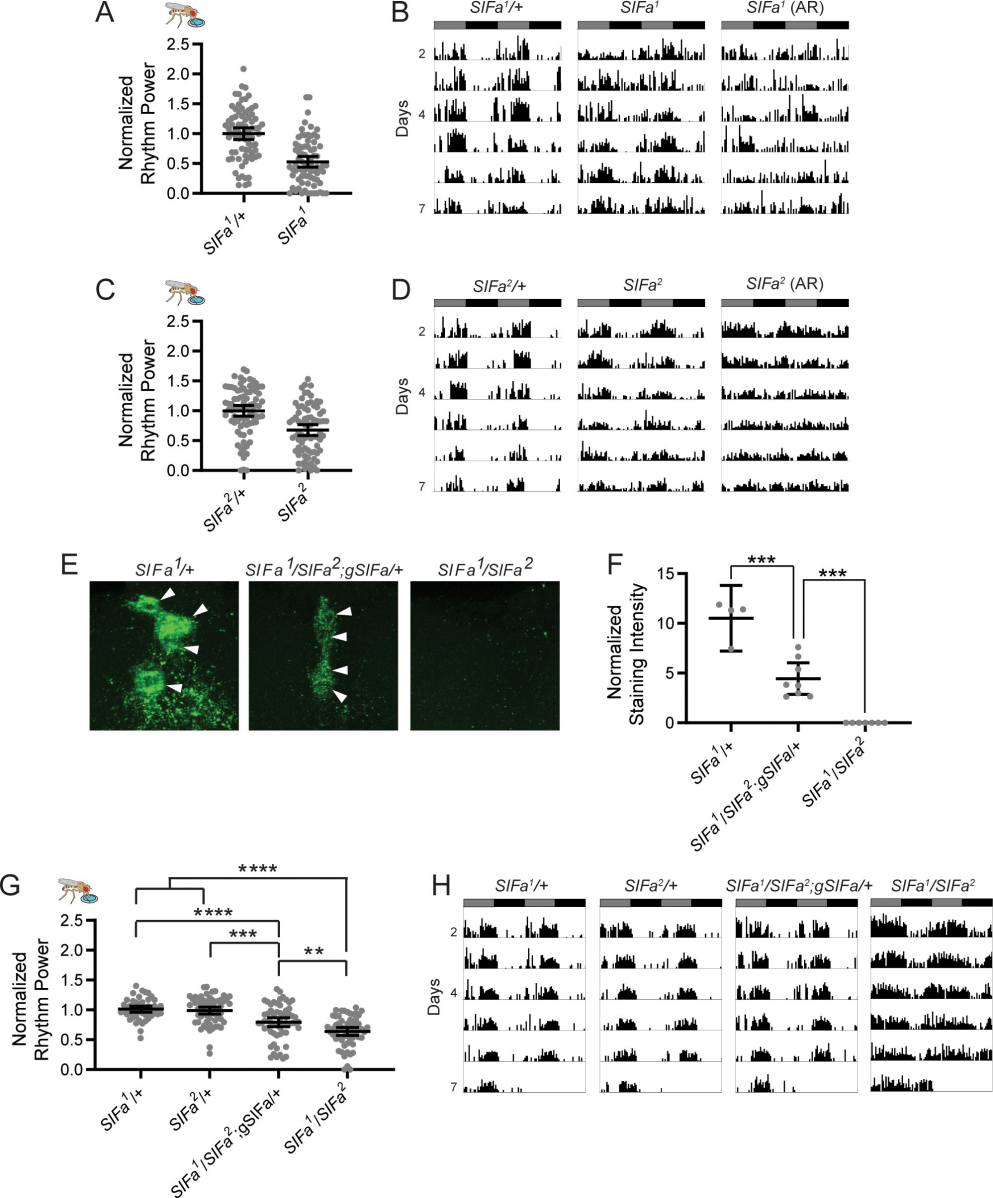
*SIFa* mutant flies have weakened feeding:fasting rhythms. (A-D) Both CRISPR/Cas9-generated *SIFa* mutant fly lines, *SIFa*^*1*^ (A-B) and *SIFa*^*2*^ (C-D), have significantly reduced feeding rhythm strength compared to heterozygous controls. (A and C) Normalized feeding rhythm power is graphed for the indicated genotypes. Dots represent strength of individual fly feeding rhythms and lines represent mean ± 95% confidence interval. (B and D) Representative single-fly feeding records are shown for experimental days 2–7 for the indicated genotypes. Flies were transferred to DD conditions at the start of experimental day 2. Feeding records show number of feeding events in 30 min bins, and data are double plotted, with each line representing two days of data. Gray and black bars represent subjective day and night, respectively. For *SIFa*^*1*^ (B) and *SIFa*^*2*^ (D) mutant flies, two records are shown: one for a fly that exhibited a rhythm power consistent with the group mean, and another for one of the ~18% of *SIFa*^*1*^ and ~10% of *SIFa*^*2*^ flies that were arrhythmic (AR). (E) Representative maximum projection confocal images of SIFa antibody staining showing a close-up of the PI region of the brain for the indicated genotypes. Note strong SIFa staining in *SIFa1/+* control brains (left), compared to an absence of staining in *SIFa*^*1*^*/SIFa*^*2*^ mutants (right). Introduction of a genomic *SIFa* rescue construct into the mutant background partially restored SIFa staining levels (middle). Arrowheads indicate SIFa+ cell bodies. (F) SIFa staining intensity, normalized to background staining levels, is plotted for the indicated genotypes. Dots represent staining intensity in individual brains and lines represent mean ± 95% confidence interval. SIFamide levels were restored to ~50% of control levels in rescue flies, with no measurable SIFamide in trans-heterozygous mutant flies. n = 4–8 per group. (G-H) Reduced feeding:fasting rhythm strength of *SIFa* mutants is partially rescued by addition of an *SIFa* genomic rescue construct. (G) Feeding rhythm power is plotted as described for (A and C). (H) Representative single-fly feeding records are plotted for the indicated genotypes as described for (B and D). For all graphs, **p<0.01, ***p<0.001, ****p<0.0001, t-test, A-B, Tukey’s multiple comparisons test, F-G. See S3 Table for exact n and *p*-values.

GAL4-UAS manipulations of SIFa+ cells demonstrate that SIFa+ neurons contribute to circadian rhythms of feeding and locomotor activity; however, they do not necessarily indicate a role for SIFa peptide itself, especially since many neurons in the fly, including those that express the *SIFa* gene, co-express multiple neurotransmitters [31,32]. Furthermore, it is possible that SIFa+ cell activation represents a gain-of-function effect that does not reflect an endogenous contribution of these neurons to feeding:fasting rhythm regulation. We therefore assessed for a role of SIFa peptide using *SIFamide* mutant and RNAi lines. We tested two CRISPR/Cas9-generated null *SIFamide* mutants, *SIFa*^*1*^ and *SIFa*^*2*^, which lack the entire *SIFa* coding sequence [14]. Strikingly, the feeding phenotypes of *SIFa* mutants mirrored those produced by TrpA1-mediated activation of SIFa+ cells. Thus, both mutant lines exhibited disrupted circadian feeding:fasting rhythms characterized by a reduction in feeding:fasting rhythm strength and fewer rhythmic flies compared to controls (Fig 4A–4D; S3 Table). Flies that were trans-heterozygous for both *SIFa* mutations (*SIFa*^*1*^*/SIFa*^*2*^) also had weakened feeding:fasting rhythms (Fig 4G and 4H; S3 Table), arguing against the possibility of nonspecific phenotypes due to off-target effects of the CRISPR/Cas9 system. The mutant phenotype also does not appear to result from developmental defects associated with lack of SIFa, as we found that cell number and morphology were grossly unchanged in these animals when we used an independent marker to label the putative SIFa cells in mutant animals (S2 Fig).

To confirm the necessity of SIFamide for proper feeding:fasting rhythms, we conducted genetic rescue experiments. Importantly, immunohistochemical analysis demonstrated that addition of a single copy of an *SIFa* genomic rescue construct restored SIFa peptide levels to ~50% of heterozygous (*SIFa*^*1*^*/+)* controls. This analysis also confirmed a complete lack of SIFa staining in mutant flies (Fig 4E and 4F). Notably, feeding:fasting rhythmicity was significantly improved following restoration of SIFa expression. Thus, the reduced feeding:fasting rhythm strength observed in trans-heterozygous mutant flies was partially restored to control levels following addition of the genomic rescue construct (Fig 4G and 4H; S3 Table). Unfortunately, addition of two copies of the genomic rescue construct was lethal, likely due to insertional effects, precluding determination of whether further increasing SIFa levels would fully restore wildtype function.

**Fig 5:**
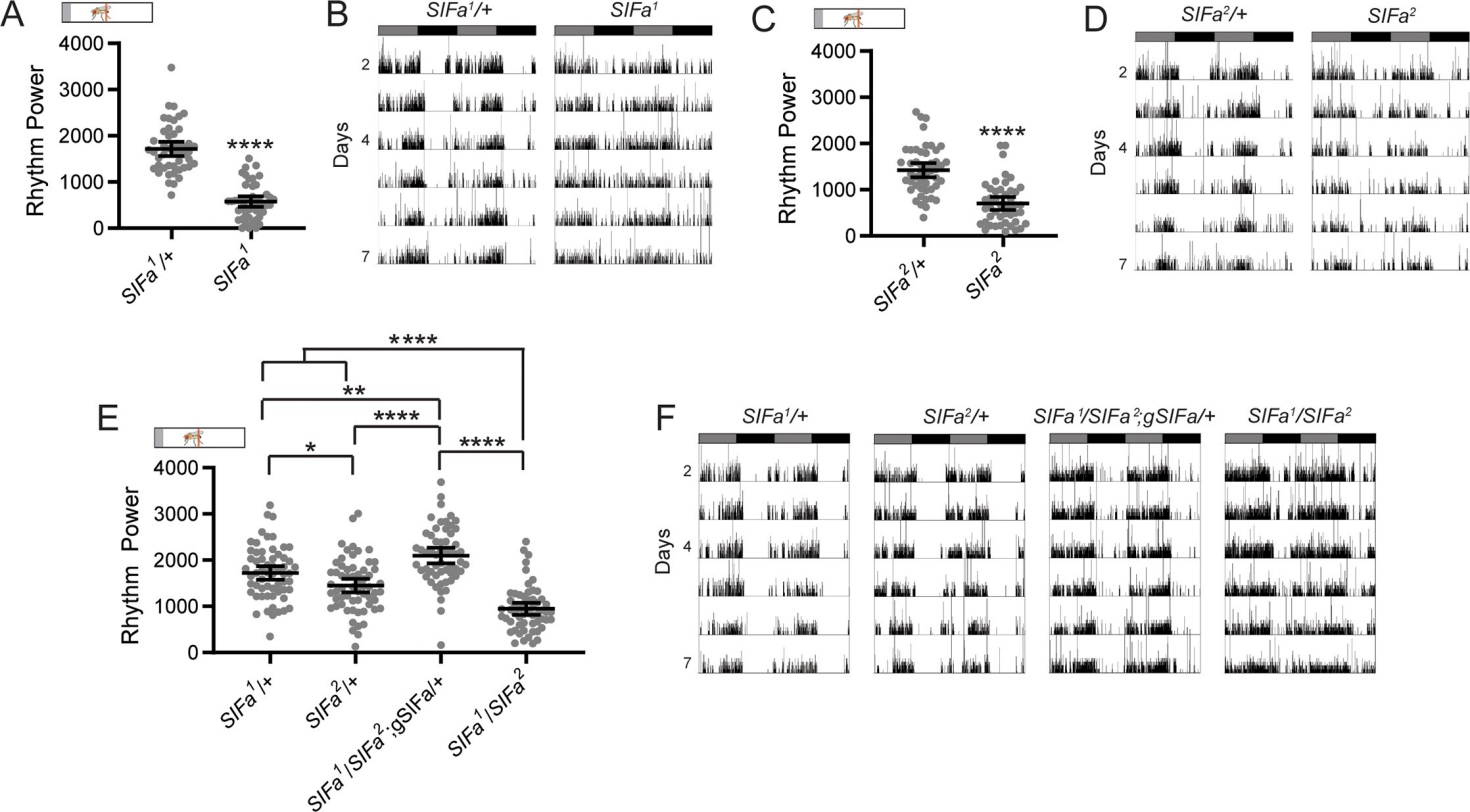
*SIFa* mutant flies have weakened rest:activity rhythms. (A-D) Both CRISPR/Cas9-generated *SIFa* mutant fly lines, *SIFa*^*1*^ (A-B) and *SIFa*^*2*^ (C-D), have significantly reduced rest:activity rhythm strength compared to heterozygous controls. (A and C) Rest:activity rhythm power is graphed for the indicated genotypes. Dots represent strength of individual fly locomotor rhythms and lines represent mean ± 95% confidence interval. (B and D) Representative single-fly activity records are shown for experimental days 2–7 for *SIFa*^*1*^ (C) and *SIFa*^*2*^ (D). Flies were transferred to DD conditions at the start of experimental day 2. Activity records show number of DAM beam breaks in 1 min bins, and data are double plotted, with each line representing two days of data. Gray and black bars represent subjective day and night, respectively. (E-F) Rest:activity rhythms of rescue flies and heterozygous control flies were significantly stronger than those of *SIFa*^*1*^*/SIFa*^*2*^ mutants. (E) Rest:activity rhythm power is plotted for the indicated genotypes as described for (A and C). (F) Representative single-fly activity records are shown for the indicated genotypes as described for (C and D), demonstrating restoration of strong rest:activity patterns in rescue flies. For all graphs, *<0.05, **<0.01, ***p<0.001, t-test, A-B, Tukey’s multiple comparisons test, E. See S4 Table for exact n and *p*-values.

Like *SIFa*>*dTrpA1* flies, *SIFa* mutants also exhibited decreased rest:activity rhythm strength (Fig 5A–5D; S4 Table). We previously reported that this phenotype was variable in mutant lines [14], but here we found it to be consistent across both homozygous and trans-heterozygous mutants, and furthermore observed complete rescue following restoration of SIFa peptide in the mutant background (Fig 5E and 5F, S4 Table). These data support a necessary role for SIFa peptide in producing robust rest:activity rhythms. It is unclear whether locomotor rhythms are directly regulated by SIFa signaling, or whether they are altered secondary to changes in feeding behavior, as feeding requires that flies are awake and active.

**Fig 6:**
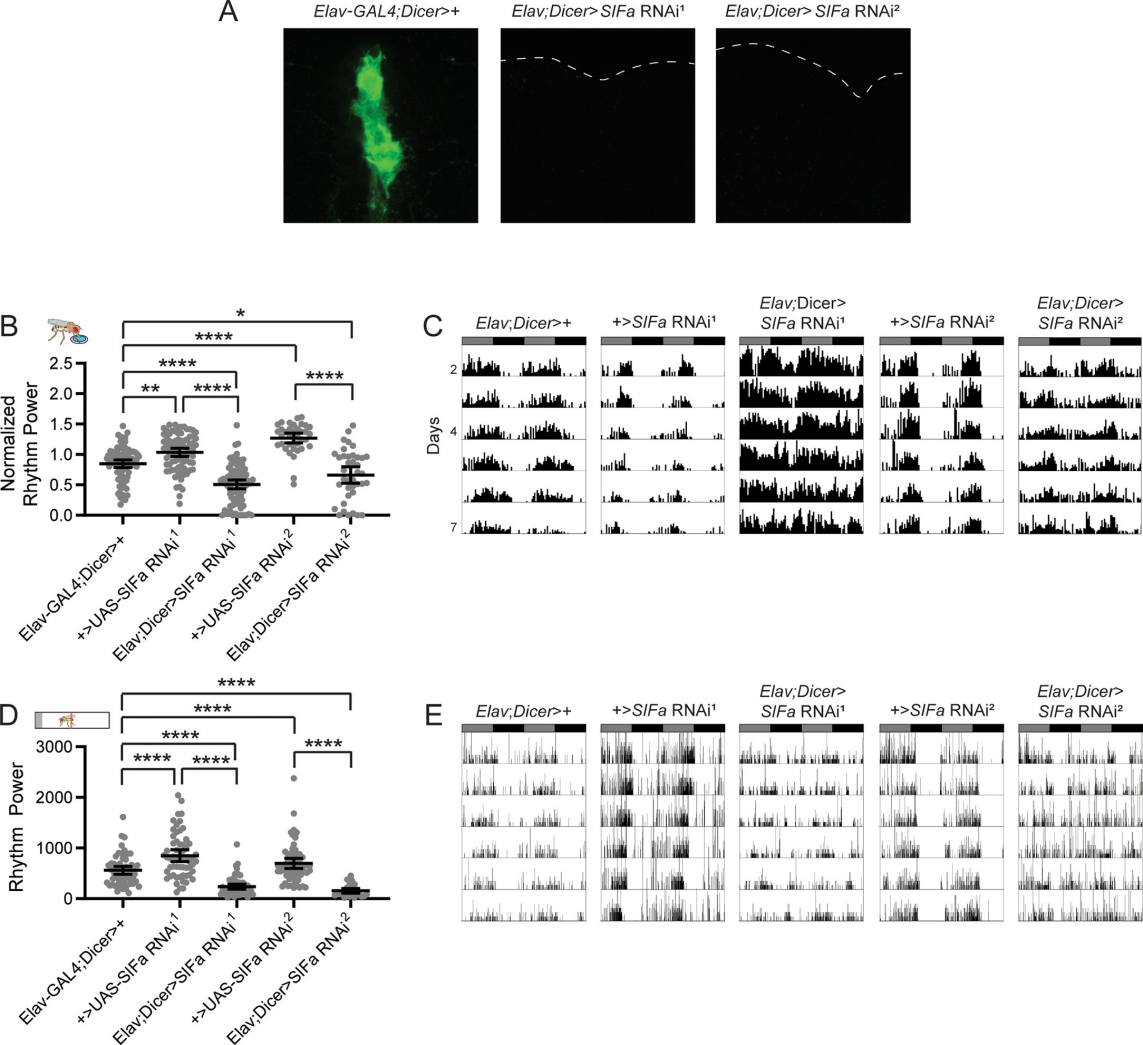
RNAi-mediated *SIFa* knockdown weakens feeding:fasting and rest:activity rhythms. (A) Representative maximum projection confocal images of SIFa antibody staining showing a closeup of the PI region of the brain for the indicated genotypes. Note strong SIFa staining in control brains (left), compared to an absence of staining in *SIFa* RNAi brains (right). Dashed lines indicate brain outline. (B) Knockdown of *SIFa* using two RNAi lines significantly reduces feeding:fasting rhythm strength. Normalized feeding rhythm power is plotted for the indicated genotypes. (C) Representative single-fly feeding records are shown for experimental days 2–7 for the indicated genotypes. Flies were transferred to DD conditions at the start of experimental day 2. Feeding records show number of feeding events in 30 min bins, and data are double plotted, with each line representing two days of data. Gray and black bars represent subjective day and night, respectively. (D) Rest:activity rhythms are significantly reduced by RNAi mediated knockdown of *SIFa*. Rest:activity rhythm power is plotted for the indicated genotypes. (E) Representative single-fly activity records are shown for experimental days 2–7 for the indicated genotypes. Flies were transferred to DD conditions at the start of experimental day 2. Activity records show number of DAM beam breaks in 1 min bins, and data are double plotted, with each line representing two days of data. For rhythm power plots, dots represent strength of individual fly normalized feeding or rest:activity rhythms and lines represent mean ± 95% confidence interval, *<0.05, **<0.01, ****p<0.0001, Tukey’s multiple comparisons test. See S3 and S4 Tables for exact n and *p*-values of feeding and locomotor results, respectively.

As an independent test of the contribution of SIFa to behavioral rhythms, we also assessed the effect of *SIFa* knockdown using two *SIFa* RNAi lines [33]. Immunohistochemical analysis revealed that both lines completely eliminated SIFa expression when driven by the pan-neuronal *Elav*-*GAL4* driver (Fig 6A). Notably, RNAi-mediated SIFa knockdown resulted in decreased feeding:fasting rhythm strength and fewer rhythmic flies as compared to genetic controls (Fig 6B and 6C; S3 Table) in a manner that phenocopied *SIFa* mutant flies. These data confirm our findings with *SIFa* mutants and provide strong evidence supporting the necessity of SIFamide for normal feeding:fasting rhythms. Both SIFa RNAi lines also exhibited significantly decreased rest:activity rhythm strength, again confirming the phenotype observed in *SIFa* mutants (Fig 6D and 6E).

### Dissociation between homeostatic and circadian regulation of feeding

**Fig 7:**
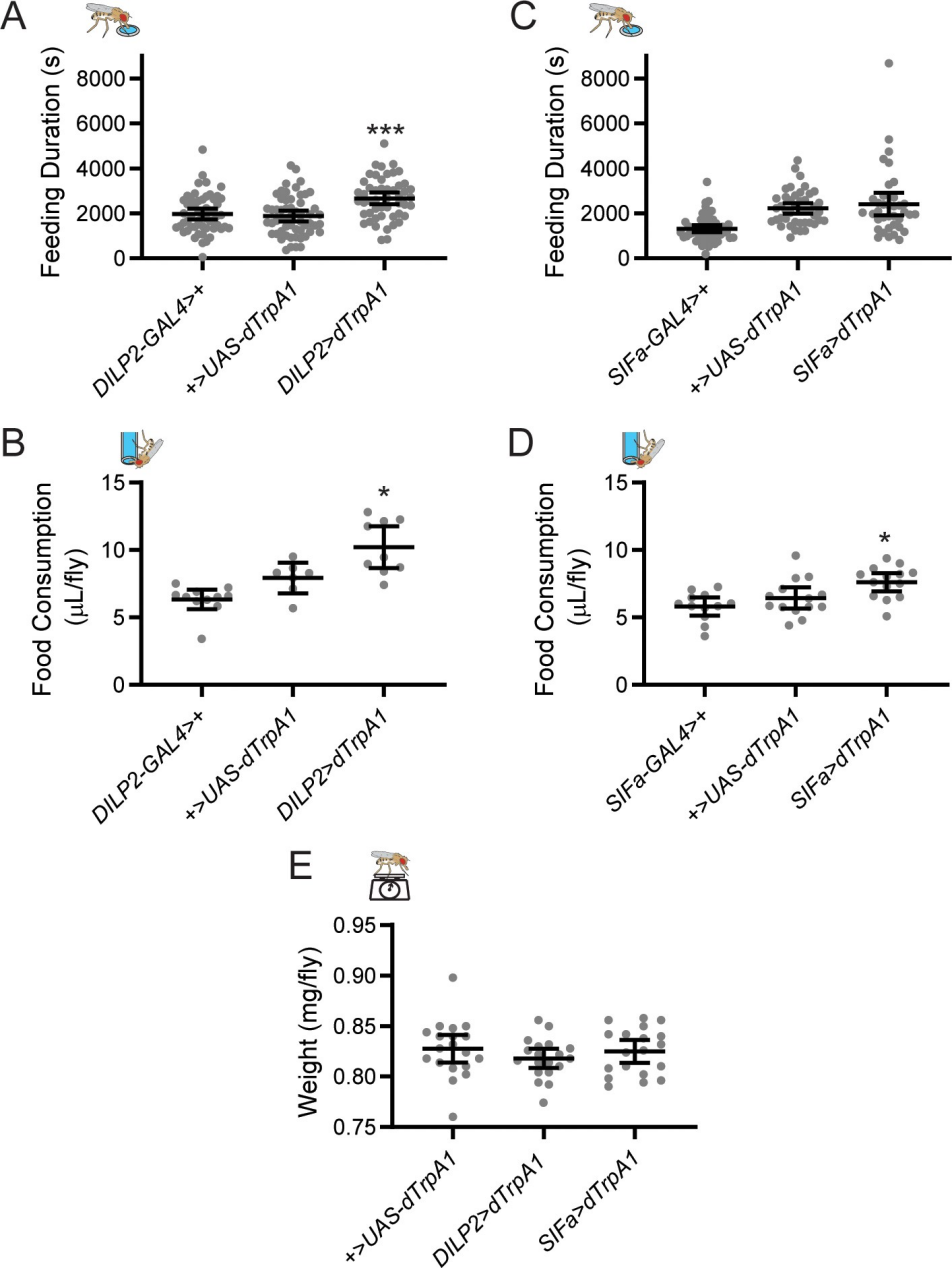
Activation of PI cell populations increases total food consumption but not overall fly weight. (A) Adult-specific dTrpA1-mediated activation of IPCs significantly increased the amount of time flies spent in contact with food in FLIC monitors over the course of a 6-d experiment compared to genetic controls. (B) IPC activation also increased the volume of liquid food consumed over a one-day period, as measured by CAFE assay. (C) Activation of SIFa+ cells had no effect on feeding duration in FLIC monitors (C) but did significantly increase the volume of food consumed as measured by CAFE assay (D). (E) Despite differences in feeding profiles across genotypes, there was no significant difference in fly weights after feeding on solid 10% sucrose food for 6 d. For all graphs, dots represent individual fly data and lines are means ± 95% confidence interval. *p<0.05, ***p<0.001, Tukey’s multiple comparisons test for experimental cross compared to both control lines.

FLIC monitoring over multiple days allowed us to identify rhythmic patterns of feeding:fasting behavior, but FLIC can also be used to assess overall feeding duration, measured as time spent in physical contact with the liquid food, which correlates strongly with food consumption [24]. Interestingly, such an analysis indicated that though feeding:fasting rhythms were unaltered in *DILP2*>*dTrpA1* flies, they spent significantly more time in contact with food than genetic controls (Fig 7A). This suggests that *DILP2*>*dTrpA1* flies have increased food intake, which we independently confirmed through the CAFE assay [34], demonstrating increased feeding volume over a 24-hr period (Fig 7B).

*SIFa*>*dTrpA1* flies also showed evidence of increased feeding. Although we did not record a significant difference in feeding duration during FLIC experiments (Fig 7C), we did observe increased food consumption compared to genetic controls in the CAFE assay (Fig 7D); however, this increased consumption was subtler than that which resulted from IPC activation. Taken together, these results indicate that IPCs and SIFa+ cells differentially regulate homeostatic and circadian regulation of feeding. The IPCs appear to regulate total food intake independent of the timing of feeding. In contrast, SIF+ PI cells contribute to the timing of feeding. The increased food intake did not result in increased body mass for either group, as we found no differences in the body weights of *DILP2*>*dTrpA1* and *SIFa*>*dTrpA1* flies compared to controls (Fig 7E).

**Fig 8:**
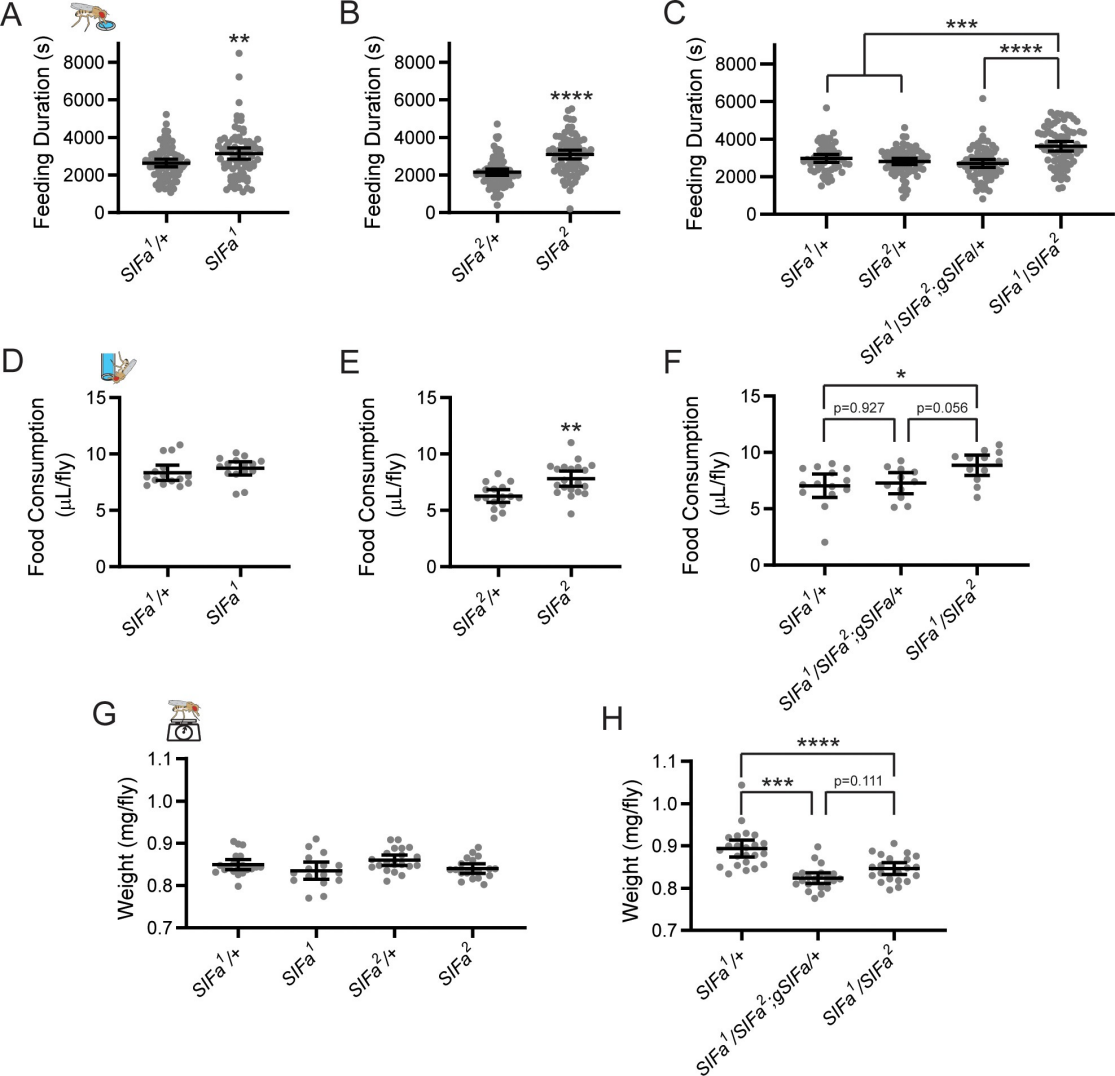
*SIFa* mutations increase total food consumption but not overall fly weight. (A-B) Both *SIFa*^*1*^ (A) and *SIFa*^*2*^ (B) mutant flies spent significantly more time in contact with liquid food in FLIC monitors over the course of a 6-d experiment compared to genetic controls. (C) Increased food interaction time in trans-heterozygous *SIFa*^*1*^*/SIFa*^*2*^ mutant flies is restored to control levels following addition of a genomic SIFa rescue construct. (D-F) Both *SIFa*^*2*^ (E) and trans-heterozygous *SIFa*^*1*^*/SIFa*^*2*^ (F) mutant flies increased the volume of liquid food consumed over a one-day period, as measured by CAFE assay. *SIFa*^*1*^ mutants (D) exhibited a non-significant trend towards increased liquid food consumption. (F) Increased feeding duration of *SIFa* mutants is partially normalized by addition of an *SIFa* genomic rescue construct. (G) Weights of homozygous *SIFa* mutant flies were not statistically different from heterozygous controls. (H) Weights of trans-heterozygous *SIFa*^*1*^*/SIFa*^*2*^ flies were significantly reduced compared to control *SIFa*^*1*^/+ controls, but this difference was not normalized by addition of an *SIFa* rescue construct. For all graphs, dots represent individual fly data and lines are means ± 95% confidence interval. *<0.05, **<0.01, ***<0.001, ****p<0.0001, t-test A-B, D-E; Tukey’s multiple comparisons test C, F-H.

We also observed evidence for altered food intake in *SIFa* mutant and knockdown flies, although as was the case for SIFa+ cell activation, the effect was not consistent across all tests. Both *SIFa*^*1*^ and *SIFa*^*2*^ homozygous mutants exhibited increased feeding duration in the FLIC assay (Fig 8A and 8B), as did trans-heterozygous *SIFa*^*1*^/*SIFa*^*2*^ flies (Fig 8C). Notably, introduction of the genomic SIFa rescue construct restored feeding duration to control levels (Fig 8C). We also observed increased feeding duration in one of the two *SIFa* RNAi lines we tested, with a trend towards increased feeding in the second (S3 Fig). In the CAFE assay, we found increased 24-hr food consumption in *SIFa*^*2*^ mutants as well as in *SIFa*^*1*^/*SIFa*^*2*^ trans-heterozygous flies, but not in *SIFa*^*1*^ flies (Fig 8D–8F). Importantly, the increased consumption in trans-heterozygous mutants was fully normalized by inclusion of the *SIFa* genomic rescue construct (Fig 8F). These data demonstrate that eliminating SIFa expression stimulates feeding behavior, but, as was the case for DILP+ and SIF+ cell activation, the increased food consumption of *SIFa* mutants occurred without a corresponding increase in fly weight. In fact, if anything, SIFa mutants tended to weigh less than controls. Though this difference did not reach statistical significance with SIFa^1^ or SIFa^2^ mutants (Fig 8G), weight was significantly reduced in trans-heterozygous SIFa^1^/SIFa^2^ mutants compared to control flies (Fig 8H). In this case, addition of the genomic rescue construct did not restore weight to control levels (Fig 8H).

### PI manipulations alter sleep and activity levels

**Fig 9:**
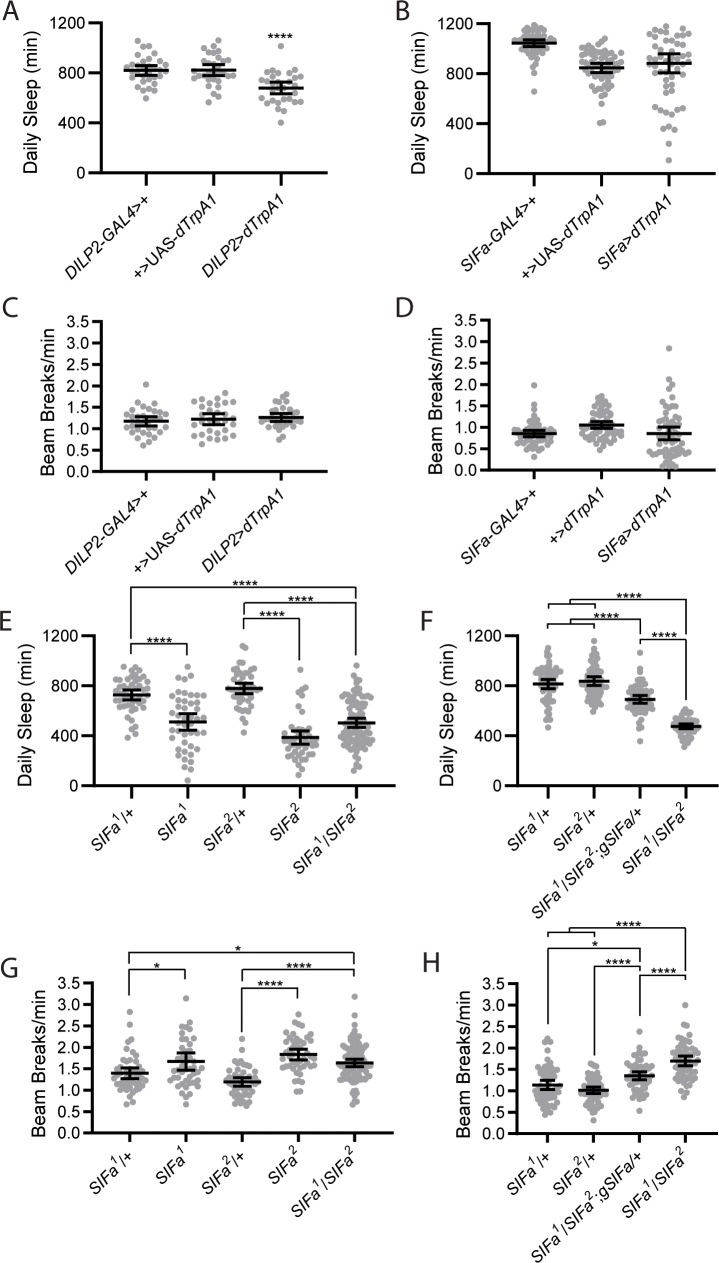
PI manipulations alter sleep and activity levels. (A) Adult-specific dTrpA1-mediated activation of DILP+ PI cells decreases total daily sleep compared to genetic controls. (B). Adult-specific dTrpA1-mediated activation of SIFa+ PI cells does not affect mean total daily sleep but increases inter-animal variability. (C) Adult-specific dTrpA1-mediated activation of DILP+ PI cells does not alter activity levels. (D) Adult-specific dTrpA1-mediated activation of SIFa+ PI cells does not affect mean activity levels but increases inter-animal variability. (E) All *SIFa* mutant flies, including both homozygous and trans-heterozygous mutants, had significantly reduced amounts of daily sleep compared to their genetic controls. (F) Reduced daily sleep of trans-heterozygous *SIFa*^*1*^*/SIFa*^*2*^ was partially restored to control levels by addition of an *SIFa* genomic rescue construct. (G) *SIFa* mutant flies exhibit significant increases in daily activity levels. (H) Increased activity of trans-heterozygous *SIFa*^*1*^*/SIFa*^*2*^ mutants was partially restored to heterozygous control levels by addition of an *SIFa* genomic rescue construct. For all graphs, mean total daily sleep or mean infrared beam breaks/min over the course of 5 consecutive experimental days is graphed. Dots represent individual fly data and lines are group means ± 95% confidence interval. *p<0.05, ****p<0.0001, Tukey’s multiple comparisons test.

Several of our manipulations resulted in increased food intake without producing overweight flies. Given that weight gain is determined by the balance between energy intake and energy expenditure [35], one possible explanation for this discrepancy could be that there is a concurrent increase in energy expenditure following our PI cell manipulations. We therefore measured daily sleep amounts to determine whether the balance of sleep and wake was altered in our experimental flies, as we reasoned that increased wakefulness would result in increased metabolic demand. Consistent with previous results [36], we found that IPC activation significantly decreased total sleep amount (Fig 9A), although there was no drastic change in sleep timing, as rest:activity rhythms were unaltered (Fig 2B). Although there was no overall effect of SIFamidergic cell activation on sleep, we did observe that sleep amounts were highly variable in *SIFa*>*dTrpA1* flies, with some showing greatly reduced sleep amount and others showing elevated sleep (Fig 9B). We also quantified activity levels following DILP+ and SIFa+ manipulations. Interestingly, there were no differences in the total daily activity between IPC activated flies and their controls (Fig 9C) despite the fact that these flies sleep less. This indicates that DILP+ cell activation alters the allocation of activity bouts such that there are fewer periods of extended inactivity. SIFa+ cell activation also failed to alter overall activity levels, but, as with sleep, increased inter-fly variability (Fig 9D).

Finally, *SIFa* mutants also showed reduced sleep amount (Fig 9E), similar to recent studies [14,22], and this was significantly ameliorated through genetic rescue (Fig 9F), which provides unequivocal evidence that SIFa is necessary for normal sleep amounts. Consistent with this finding, *SIFa* mutants also showed a corresponding increase in overall daily activity that was partially normalized via inclusion of the genomic rescue construct (Fig 9G and 9H). These flies were not hyperactive, however, as their mean activity during wake periods was unchanged compared to controls (S4 Fig). Taken together, the reduction in sleep concomitant with the increased activity levels that we observed across multiple SIFa manipulations may explain the lack of weight gain in these flies, despite their increased food consumption.

### SIF+ PI Cells communicate broadly throughout the brain

Previous studies have demonstrated distinct projection patterns of DILP+ and SIFa+ PI neurons. The IPCs have been reported to have relatively simple projections that primarily innervate neurohemal organs via the esophageal canal, as well as directly releasing DILPs into the hemolymph [18]. In addition, axon collaterals are present in the tritocerebral area of the brain just ventral to the esophageal canal [37,38]. In contrast, SIFa+ cells have been shown to extend axons widely throughout the brain [12,21,23]. To expand upon these findings, we used the *DILP2*- and *SIFa*-*GAL4* lines to drive expression of genetic markers that allowed us to separately label dendritic and axonal neuronal compartments and to assess synaptic connectivity.

**Fig 10:**
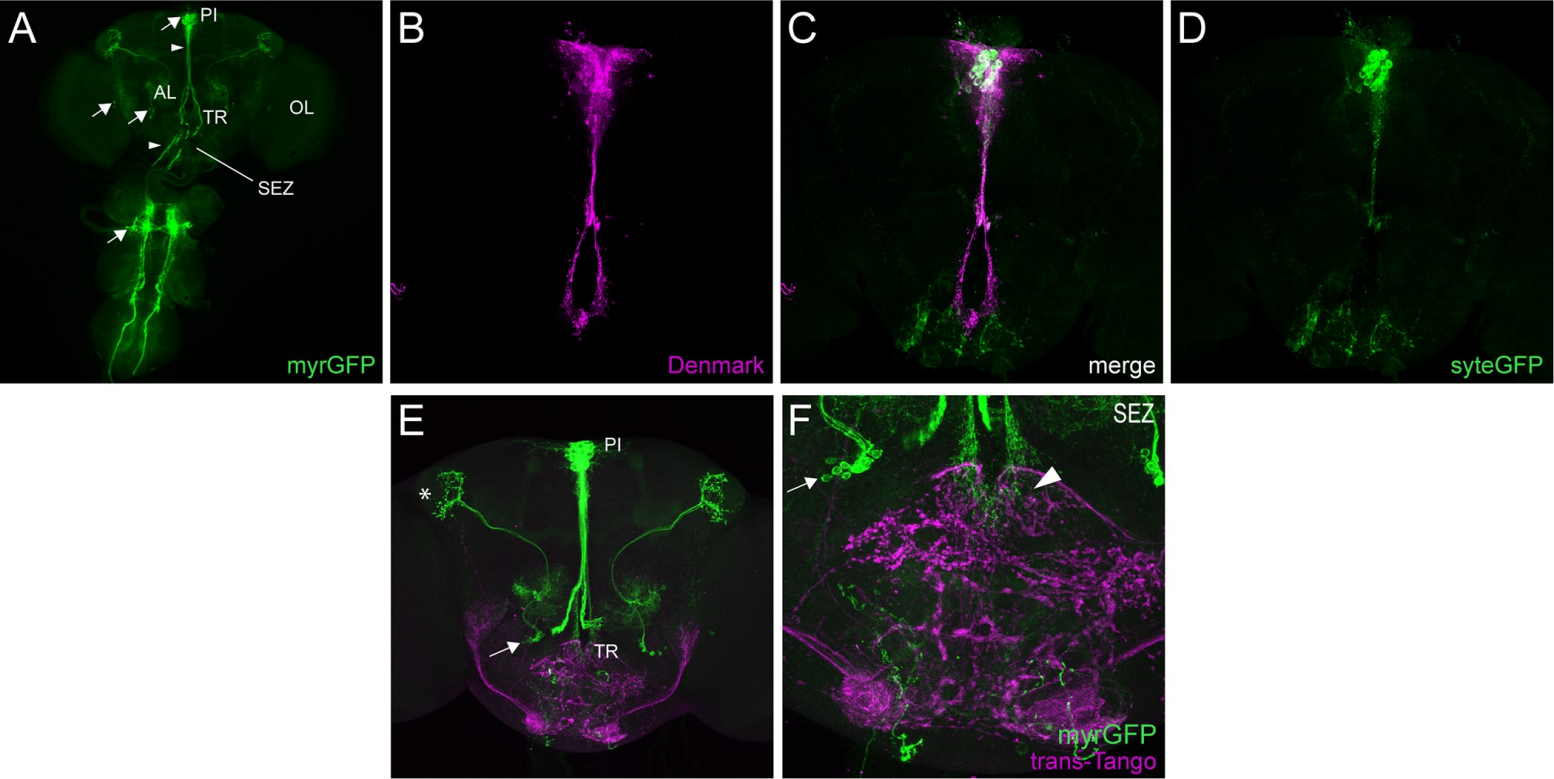
IPCs make limited synaptic contacts with other brain regions. (A) Representative maximum projection confocal image of a brain and ventral nerve cord in which *DILP2*-*GAL4* was used to drive expression of myristoylated GFP (myrGFP), which is visualized with GFP antibody staining. Arrows point to *DILP2*-*GAL4*+ cell bodies. Strong *DILP2*-*GAL4* expression is found in ~14 PI cells (top arrow) and a paired group of cells in the ventral nerve cord (bottom arrow), with additional lower-level expression in two groups of cells located lateral to the esophageal canal (middle arrows). Arrowheads point to the ventral projections of the *DILP2*-*GAL4+* PI cells, which extend towards (upper arrowhead) and duck into (lower arrowhead) the esophageal canal, and also terminate in the tritocerebral area (TR) of the subesophageal zone (SEZ). (B-D) Representative maximum projection confocal image of a brain in which *DILP2*-*GAL4* was used to simultaneously drive expression of the dendritically-localized Denmark (magenta), which is visualized with RFP antibody staining (B), and the axonally-localized synaptotagmin-eGFP (sytEGFP, green), which is visualized with GFP antibody staining (D). A merged image is shown in (C). The ventral projections of *DILP2*-*GAL4*+ PI cells contain both axonal and dendritic compartments. (E) Representative maximum projection confocal image of a brain in which *DILP2*-*GAL4* was used to simultaneously drive expression of trans-Tango (magenta), visualized with a HA antibody staining, which identifies postsynaptic cells, and myrGFP (green), which labels the *DILP2*-*GAL4*+ cells. trans-Tango signal as restricted to the SEZ region just ventral to the esophagus. The arrow indicates non-PI cells expressing *DILP2*-*GAL4*, which arborize in the lateral horn, which is marked by the asterisk. (F) Close-up image from the brain shown in (E) demonstrating trans-Tango signal in the SEZ. The arrowhead indicates trans-Tango signal in the tritocerebrum adjacent to the descending axons of the DILP2+ PI cells. The arrow indicates non-PI cells expressing *DILP2*-*GAL4*.

We first used the *DILP2-GAL4* line to drive a membrane-targeted myristoylated GFP (myr-GFP), which labels cell bodies, axons and dendrites. Analysis of these flies confirmed strong *DILP2*-*GAL4* expression in ~14 PI cells with neuronal processes extending ventrally towards the esophageal canal (Fig 10A). We found additional, low-level GAL4 expression in 2 clusters of cells that are positioned lateral to the esophageal canal, as well as in a small group of neurons in the thoracic ganglion of the ventral nerve cord (Fig 10A). These non-PI cell clusters have also been observed previously [38]. When we used the same GAL4 line to drive concurrent expression of synaptotagmin-eGFP (syt-eGFP) [39], which is targeted to axon terminals, and Denmark [40], which preferentially labels dendrites, we found that the ventral projections of the IPCs contain a mix of input and output sites (Fig 10B–10D). We observed very little signal in other areas of the brain, consistent with the limited projection pattern of these cells. There was syt-eGFP signal throughout the subesophageal zone (SEZ); however, it is likely at least some of this signal derives from ascending projections from the intrinsic ventral nerve cord neurons labeled by *DILP2*-*GAL4*. Finally, we characterized downstream synaptic connections of IPCs to other regions of the brain and ventral nerve cord using trans-Tango [25]. While unable to definitively mark downstream signaling partners affected by paracrine signaling of neurosecretory cells, trans-Tango does provide a clear visualization of synaptic connectivity within the brain. As expected based on the distribution of syt-eGFP+ processes, trans-Tango signal was largely limited to the tritocerebral area just below the esophagus (Fig 10E and 10F), with additional trans-Tango in the SEZ likely arising from non-PI *DILP2*-*GAL4*+ cells.

**Fig 11:**
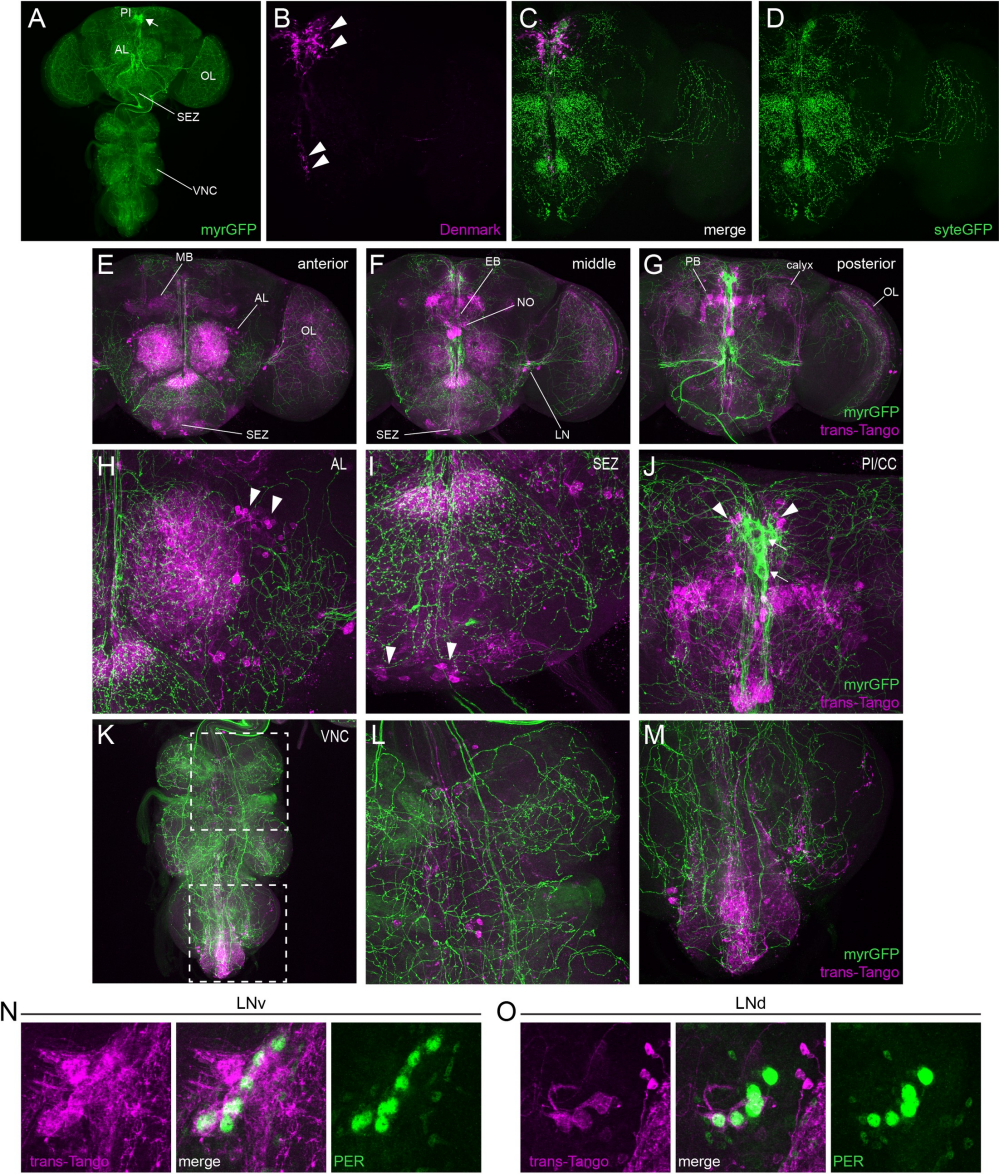
SIFa+ cells have extensive connectivity throughout the brain and ventral nerve cord. (A) Representative maximum projection confocal image of a brain and ventral nerve cord in which *SIFa*-*GAL4* was used to drive myristoylated GFP (myrGFP), which is visualized with GFP antibody staining. Arrow points to 4 *SIFa*-*GAL4*+ cell bodies in the PI region of the brain. Projections of these cells extend throughout the brain including to the antenna lobe (AL), optic lobe (OL), and subesophageal zone (SEZ), and also into the ventral nerve cord (VNC). (B-D) Representative maximum projection confocal image of a brain in which *SIFa*-*GAL4* was used to simultaneously drive expression of the dendritically-localized Denmark (magenta), which is visualized with RFP antibody staining (B), and the axonally-localized synaptotagmin-eGFP (sytEGFP, green), which is visualized with GFP antibody staining (D). A merged image is shown in (C). Dendritic compartments are concentrated around the PI (upper arrowheads in B), with some descending projections towards the esophagus (lower arrowheads in B). Axonal compartments are localized throughout the brain. (E-G) Representative confocal stacks showing the anterior (E), middle (F) and posterior (G) sections of a brain in which *SIFa*-*GAL4* was used to simultaneously drive expression of trans-Tango (magenta), visualized with a HA antibody staining, which identifies postsynaptic cells, and myrGFP (green), which labels the SIFa+ cells. trans-Tango signal is found in cells throughout the brain, including AL, SEZ, OL, lateral circadian clock neurons (LN), the mushroom body (MB, with cell bodies in the calyx), and several areas of the central complex such as the ellipsoid body (EB), fan-shaped body, protocerebral bridge (PB), and noduli (NO). (H-J) Close-up images from the brain shown in (E-G) demonstrating trans-Tango signal (magenta) in neurons projecting into the AL (H, arrowheads), SEZ (I, arrowheads), and central complex (J, arrowheads). For E-J, myrGFP (green) labels SIFa+ cell bodies and processes. Note the SIFa+ cell bodies in the PI region, just dorsal to the central complex (J; arrows). (K-M) Representative maximum projections confocal images of the VNC of flies in which *SIFa*-*GAL4* was used to drive trans-Tango (magenta) and myrGFP (green). (L-M) show close-ups of the boxed regions in K. Note that SIFa+ neurites ramify throughout the VNC, with trans-Tango+ cell bodies in thoracic and abdominal ganglia. (N-O) Representative confocal stacks showing the ventrolateral (LNv) (N) and dorsolateral (LNd) (O) core clock neurons of flies in which *SIFa*-*GAL4* was used to drive trans-Tango (magenta). Brains were also stained for the presence of the PERIOD protein (green). We consistently observed colocalization between these two markers, demonstrating that these groups of core clock neurons are postsynaptic to SIFa+ cells.

A strikingly different picture emerged when we conducted similar analyses to investigate the connectivity of SIFa+ cells. When we used *SIFa*-*GAL4* to drive expression of myr-GFP, we confirmed previous reports demonstrating extensive ramifications of SIFa+ processes throughout the brain and ventral nerve cord (Fig 11A). The dendrites of these neurons are concentrated in the PI region, close to the cell bodies, as well as on projections that course ventrally towards the esophagus (Fig 11B). In contrast, syt-eGFP+ axon terminals are distributed throughout the brain (Fig 11D), which suggests that SIFamidergic neurons communicate with a vast network of downstream neuronal populations. Importantly, trans-Tango analysis demonstrated that this is indeed the case. We found trans-Tango signal present in post-synaptic neurons in all regions in which syt-eGFP+ axons are present. This includes areas that have previously been implicated in feeding regulation such as the antennal lobes (AL) (Fig 11E and 11H), which receive olfactory information and have been shown to respond to thermogenetic activation of SIFa+ cells [23,41], and the SEZ (Fig 11E, 11F and 11I), which receives sensory information about taste and contains motor and interneurons involved in feeding control [19,42]. Further experiments are necessary to determine the identity of these postsynaptic cells to assess whether they correspond to neurons that have been directly implicated in feeding regulation.

In addition to the feeding-related areas, we observed prominent trans-Tango signal in cells that project into the mushroom body (Fig 11E and 11G), multiple regions of the central complex (Fig 11F, 11G and 11J), the optic lobes (Fig 11E–11G), and the ventral nerve cord (Fig 11K and 11M). Finally, and somewhat surprisingly, trans-Tango signal was present in several groups of core clock neurons, the identity of which was confirmed by presence of the PERIOD protein, which is a central component of the molecular clock (Fig 11N and 11O). This implicates SIFamidergic output cells in feedback control of the core clock network and provides a potential mechanism through which food intake and energy status could alter core clock cycling.

**Fig 12:**
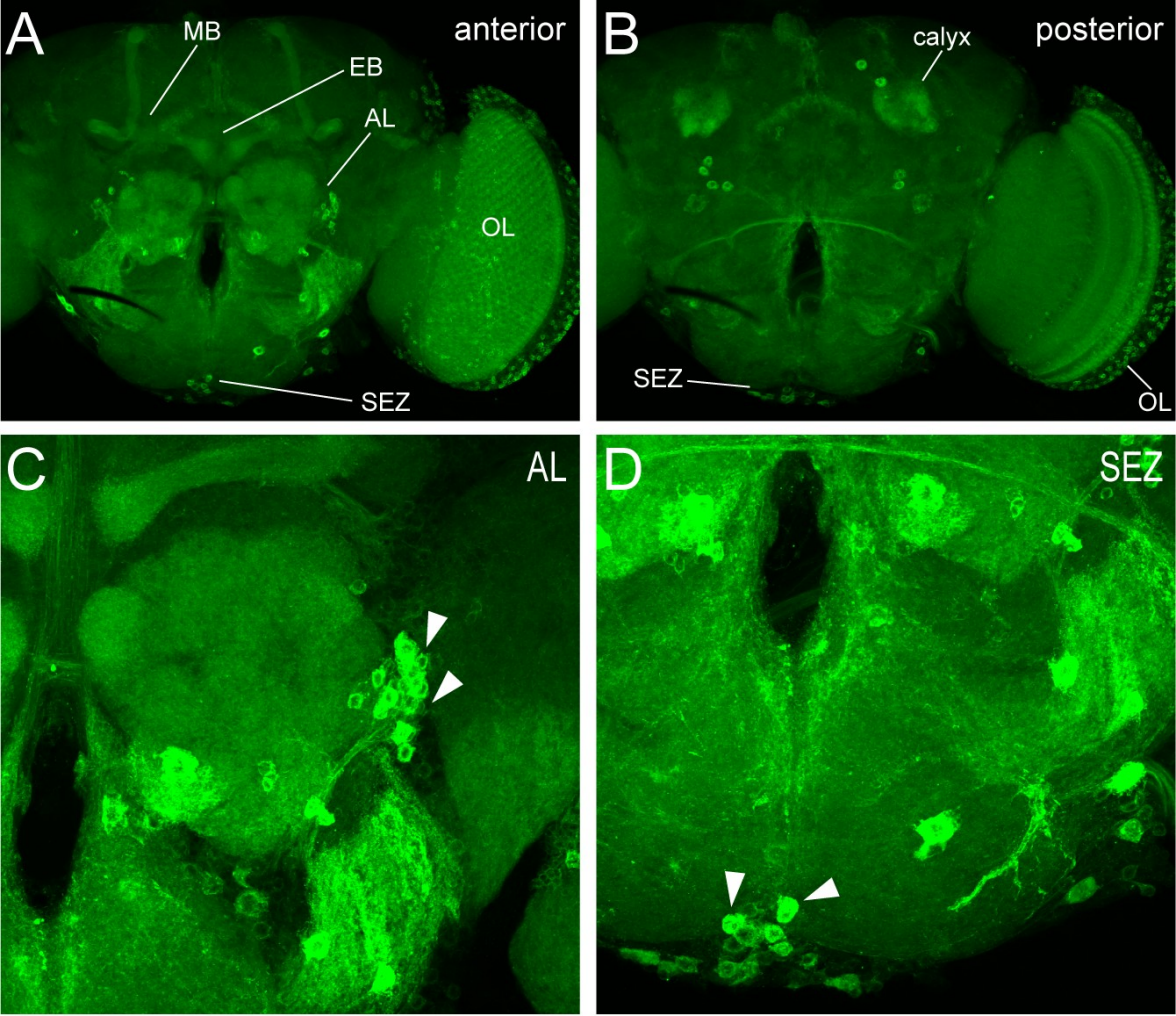
SIFa receptor (SIFaR) expression is consistent with trans-Tango analysis of SIFa+ cell synaptic partners. (A-B) Representative confocal maximum projection stacks showing the anterior (A) and posterior (B) sections of a brain in which *SIFaR*-*GAL4* was used to drive expression of CD8:GFP (green), which labels the SIFaR+ cells. *SIFaR*-*GAL4* expression is found in cells throughout the brain, with particularly bright signal in cells in the OL, SEZ, and cells projecting into the MB, EB and AL. (C-D) Close-up images from the brain shown in (A-B) demonstrating SIFaR expression in neurons projecting into the AL (C, arrowheads) and SEZ (D, arrowheads).

To further investigate the extensive connectivity of SIFa+ cells throughout the brain, we mapped SIFa receptor (SIFaR) expression using a knock-in GAL4 [43]. We found substantial overlap in expression of SIFaR and the most concentrated regions of trans-Tango signal including in the MB, AL, OL, and SEZ (Fig 12A–12D). The widespread expression of SIFaR confirms the presence of multiple downstream signaling partners of SIFamide in the brain and demonstrates that SIFa peptide is capable of acting on these downstream targets.

## Discussion

At its core, the circadian system is made up of central clock neurons in the brain that keep time through the presence of cell-autonomous molecular clocks. To enact behavioral rhythms, these clock cells must be connected through output pathways to downstream neuronal populations that directly control behavioral outputs [7,9]; therefore, a complete understanding of circadian regulation of behavior depends on the delineation of output circuitry. Here we identified a population of SIFa+ neurons in the pars intercerebralis that comprises part of the output pathway controlling feeding:fasting rhythms in flies. Constitutive activation of these cells strongly compromises normal patterns of feeding behavior, including producing a substantial percentage of flies that feed arrhythmically. We also pinpointed a specific contribution of SIFa peptide to feeding rhythms, as *SIFa* mutant and RNAi knockdown lines show similar reductions of feeding rhythm strength.

The identification of a neuronal population and associated signaling molecule for the control of feeding:fasting rhythms should facilitate future studies aimed at further dissecting feeding output circuits, with the ultimate aim of tracing the pathway to motor neurons that directly control feeding. To that end, our trans-Tango analysis demonstrated that many neurons throughout the brain are postsynaptic to SIFa+ PI cells, including in areas such as the AL, which is involved in olfactory processing, and the SEZ, which is involved in gustatory processing and also contains feeding-related motor neurons [19,41,42]. It will be of interest to more definitively determine the functional and neurochemical identity of postsynaptic neurons and to assess whether manipulations of SIFa receptor expression in these putative downstream output cells can recapitulate the feeding phenotypes observed following SIFa+ cell manipulations.

A role for SIFa in feeding regulation is supported by a recent study that demonstrated that SIFa modulates olfactory processing under conditions of starvation [23]. Flies normally show sensitized AL projection neuron responses to food odors following starvation, however, this sensitization is absent in flies in which *SIFa* expression has been reduced through RNAi mechanisms. Martelli et al. (2017) also showed that SIFa+ cells exhibit increased activity in response to starvation, and that thermogenetic activation of SIFa+ cells increases food consumption in satiated flies. Their experiments suggest that SIFa tunes sensory responsiveness to food cues according to the energy status of the fly, which subsequently increases feeding propensity in energy-depleted states. The described effects here identify an additional function of SIFa in dictating temporal patterns of feeding.

Interestingly, although our findings of increased food consumption following SIFa+ cell activation are in line with those of Martelli et al. (2017), we found that feeding amount was also elevated in *SIFa* mutant flies, which is not predicted by a model in which SIFa peptide solely serves to increase appetitive and feeding behavior. This suggests that the exact nature of the regulation of feeding by SIFa is complex and may vary depending on environmental conditions and internal state. It is unclear why food consumption would be similarly affected by manipulations that eliminate SIFa peptide and those that hyperactivate SIFa+ cells, which should result in heightened SIFa signaling. One possibility is that constitutive SIFa+ cell activity could ultimately deplete SIFa stores, thus mimicking the *SIFa* mutant phenotype. Alternatively, feeding phenotypes may be affected differentially by *SIFa* mutations, which are present throughout development, compared to adult-specific thermogenetic activation. Regardless of whether acute SIFa signaling stimulates or inhibits food consumption, the fact that SIFa+ cell activation and reduction of *SIFa* signaling via mutations, cell ablation, or RNAi knockdown consistently degrade feeding:fasting rhythms provides strong evidence for a central contribution to the determination of the timing of feeding.

Flies in which SIFa+ cells are constitutively activated or that lack SIFa peptide due to cellular ablation or mutation also exhibit significantly reduced rest:activity rhythms, which is consistent with previous results demonstrating weakened locomotor rhythms following ablation of these cells [12]. This effect was most pronounced in *SIFa>reaper* flies, indicating a potential for additional neurotransmitters emanating from SIFa+ cells to contribute to the regulation of locomotor activity. The overlap of feeding:fasting and rest:activity disruption raises the question of whether SIFa cells independently regulate feeding and locomotor rhythms, or whether one of these is indirectly affected secondary to changes in the other. Feeding and locomotor activity are interconnected behaviors that usually coincide, as animals primarily feed during their active phase [44]. Nevertheless, feeding and locomotor rhythms can be dissociated in both flies and mammals. For example, adipocyte-specific knockout of the mammalian clock gene *Arntl* attenuates feeding rhythms in mice while leaving rest:activity rhythms intact [45], and mutations in mammalian *per1* and *per2* genes have differential effects on the phasing of locomotor and feeding rhythms [46]. A similar phenotype has been noted in flies, as cell-specific abrogation of the molecular clock in the *Drosophila* fat body, a peripheral metabolic tissue, selectively alters the phase and magnitude of feeding rhythms without changing cycles of rest and activity [47]. More recently, it was shown that manipulations that downregulate DH44 signaling or silence neurons expressing the hugin peptide significantly degrade rest:activity rhythm strength in DD conditions but leave the strength of DD feeding:fasting intact [15]. Taken together, these results confirm that feeding:fasting rhythms are under *de facto* circadian control and do not simply occur secondary to rest:activity rhythms. Because locomotor rhythm disruption can occur independent of changes in feeding behavior, we conclude that the effects of our SIFa manipulations likely reflect direct feeding:fasting rhythm regulation.

In addition to affecting rest:activity and feeding:fasting rhythms, adult-specific SIFa+ cell manipulations also resulted in high lethality, particularly in the case of adult-specific neuronal silencing. This suggests that SIFa+ cells perform some necessary function in the adult animal, though it seems that SIFa peptide itself is dispensable for survival, as mutants eclose at expected Mendelian ratios. Intriguingly, we found that a substantial number of flies eclosed from genetic crosses that result in SIFa+ cell ablation during developmental stages due to expression of the apoptotic gene *reaper*. The lack of a lethality phenotype in SIFa+ ablated flies is perhaps due to compensatory changes in these flies that are not present following adult-specific manipulations. It is unclear whether the lethality phenotype is related to alterations in feeding behavior following SIFa+ cell manipulations, or whether other, yet unidentified contributions of SIFa+ cells are responsible, but as there is little evidence for SIFa expression in cells outside of the PI, it is likely that the phenotype stems from dysregulation of these cells.

Together with previous findings, the current results add to a growing understanding of the PI in the control of circadian outputs. The PI is situated in a region of the *Drosophila* brain that is near the axon terminals of multiple groups of core clock cells [48], and previous work has shown anatomical and functional connections between clock cells and multiple PI populations, including those expressing DH44, SIFa and DILPs [12,16,17]. These clock cell inputs could allow PI cells, which lack molecular clocks, to transmit circadian information to downstream output regions. Interestingly, the PI cell populations appear to differentially contribute to circadian outputs. As detailed above, DH44+ cells selectively regulate rest:activity rhythms while SIFa+ cells contribute to both rest:activity and feeding:fasting rhythms. DILP+ PI cells contribute to neither behavioral rhythm but instead have been shown to modulate circadian gene expression in the fat body [17]. These results support the hypothesis that the PI is a circadian output hub that channels core clock input into anatomically distinct output pathways to coordinately regulate different circadian outputs.

Though we found no effect of DILP+ cell manipulations on feeding:fasting rhythms, we did observe changes in overall food intake following IPC activation in two independent assays, which is consistent with a homeostatic role for these cells. The IPCs receive feedback from a range of circulating peptides and are also indirectly targeted by satiety signals secreted from the fat body [49,50] integrating information regarding the nutritional status of a fly as one component of the intricate regulation of energy homeostasis [38]. DILP+ neurons have also recently been shown to play a role in nutrient sensing in female flies, contributing to the modulation of reproductive dormancy by affecting overall feeding and maintaining females in a metabolically active state [51]. Generally, IPC neuronal activity is regulated by feeding status, as the cells are more active in the fed versus starved state [52], which likely results in increased DILP secretion in fed flies [53]. In turn, insulin/IGF signaling (IIS) is an integral regulator of growth and development and affects a range of physiological attributes including metabolism, reproduction, stress response, and aging [54].

DILPs have also been directly implicated in regulating feeding behavior, with several studies demonstrating anorexigenic effects of increased DILP signaling [55–58], as well as of drosulfakinin peptides, which are an additional output of the IPCs and act as a satiety signal [59]. These effects are in line with evidence demonstrating increased activation of IPCs and release of DILPs in the fed state. In contrast, it has also been shown that DILP+ cell silencing can result in hypophagia, as indicated by reduced fecal output [60], and that thermogenetic DILP+ cell activation can either stimulate or inhibit feeding depending on metabolic status [61]. Thus, the role of the DILP+ PI cells in determining overall food consumption, similar to SIFa peptides, is likely complex [19]. Given this, our finding of increased feeding following IPC stimulation, though counterintuitive, is not without precedent, and may occur as a result of an interaction between diet type, insulin signaling, and the metabolic condition of the flies, especially as they are exposed to a carbohydrate-only diet in our FLIC and CAFE assays. Alternatively, increased feeding could occur if DILPs are depleted by extended IPC activation. This possibility could be directly tested using recently-developed DILP2 reporter flies [54], which allow for sensitive measurements of circulating DILP2 levels.

The contrasting effects of DILP+ and SIFa+ PI cell activation demonstrate dissociable control over homeostatic and circadian regulation of feeding by these two populations of PI cells. The results of our trans-Tango analyses provide a potential anatomical basis for this and suggest that DILP+ and SIFa+ PI cells rely on different signaling paradigms. Given their limited connectivity to other brain regions, the IPCs likely release DILPs systemically to act on target tissues, including the brain, via long-distance diffusion through the hemolymph [18]. In contrast, SIFamidergic cells appear to act via direct synaptic connections to impact widespread brain areas. The differences in kinetics between these two signaling mechanisms could underlie the functional differences of these cell populations with respect to feeding regulation, with IPC activity reflecting overall energy status and therefore controlling homeostatic aspects of feeding, and SIFa cells regulating moment-to-moment feeding decisions and therefore controlling circadian patterns of feeding. In addition, the downstream connections of SIFa+ cells to central clock neurons, including l-LN_v_s and s-LN_v_s as well as LN_d_s, implicates SIFa+ cells in feedback control of the central clock. We previously found no alterations in central clock timing following SIFa+ cell ablation [12]; however, as SIFa+ cells appear to lie at a crossroads of energetic signaling [23], it follows that they would have the capacity to relay that information back to the core clock and affect behavioral changes that are attuned to the circadian patterns of activity as necessary, perhaps under conditions in which food access is limited.

Research into metabolic and feeding control continues to uncover a dense web of interconnected regulators, indicative of how integral proper nutrient signaling is to overall organismal health. The partial reduction of feeding rhythm strength ascribed to SIFa here leaves room for the discovery of additional signals affecting circadian feeding rhythms. Two promising neuropeptides that have been shown to affect feeding behaviors are short neuropeptide F [62] and allatostatin A [63,64], both of which also have been associated with sleep regulation in flies [65,66]. Characterizing the complete output circuit of circadian feeding behavior in flies will help identify the most important contributors to synchronized feeding patterns and increase our understanding of the profound metabolic consequences of circadian disruption.

## Materials and methods

### Fly lines

The following fly lines were used: Iso31 (isogenic w^1118^) [67], *SIFa*-*GAL4* [21], *DILP2*-*GAL4* (FBti0147109) [26], *Elav*-*GAL4*; UAS-*Dicer2* (RRID:BDSC_25750), *SIFaR*-*GAL4* [43], UAS-myrGFP,QUAS-mtdtomato-3xHA; trans-Tango (RRID:BDSC_77124) [25], UAS-mCD8:GFP (RRID:BDSC_5137), UAS-*dTrpA1* (FBti0114501) [27], UAS-*reaper* (RRID:BDSC_5823), UAS-*SIFa* RNAi^1^ (RRID:BDSC_29428), UAS-*SIFa* RNAi^2^ (RRID:BDSC_60484), UAS-*Kir2*.*1* (FBti0017552), and *tub*-GAL80^TS^ (FBti0027796) [29]. The latter two stocks were combined to create *tub*-GAL80^TS^; UAS-*Kir2*.*1* flies (referred to as UAS-*Kir2*.*1*^TS^), which were used for temperature-dependent neuronal silencing. *SIFa*^*1*^ and *SIFa*^*2*^ mutants [14] were generated via CRISPR/Cas9 genome editing and lack the entire SIFa coding sequence. Mutant lines were outcrossed 7 generations to the iso31 background before testing.

### Generating the SIFa genomic rescue construct

A 3,678 bp genomic sequence containing the entire *SIFa* coding sequence including the 3’ UTR and 3,229 bp upstream of the translational start site was PCR amplified using the following primers, which also added NotI sites (underlined) for cloning into pattB (30): gSIFa_up: 5’-TATGCGGCCGCAGAGCGAGTTCAGTGCTGTA-3’; gSIFa_down: 5’-TATGCGGCCGCGCCCGAAACCGAGCCACTCG-3’. The resulting plasmid was inserted via phiC31-mediated integration into PBac{yellow[+]-attP-3B}VK00033 (RRID:BDSC_9750) by BestGene, Inc. (Chino Hills, CA). Genomic rescue flies were outcrossed 7 generations to the iso31 background before behavioral testing.

### Rest:activity rhythm and sleep analysis

For mutant and rescue experiments, flies were reared on standard cornmeal-molasses food and entrained to a 12 hr:12 hr (12:12) light-dark (LD) cycle at 25°C for at least 3 days prior to behavioral analysis. Following entrainment, 5–10 day old male flies were placed in glass tubes containing 5% sucrose/2% agar food for monitoring using the Drosophila Activity Monitoring (DAM) System (Trikinetics). Monitoring was conducted for 7 days under conditions of constant darkness (DD) and activity readings were taken every minute. Temperature sensitive dTrpA1 and GAL80^TS^ experiments were conducted as described above except flies were reared and entrained at 18°C to prevent premature activation or inhibition, and following loading into the DAM system, were transferred to constant darkness (DD) for 1 day at 18°C followed by 7 days DD at 28°C. Locomotor rhythms for individual flies were calculated using χ^2^ periodogram analysis with ClockLab software (Actimetrics) for days 1–6 of DD for flies entrained at 25°C or days 2–7 of DD for flies entrained at 18°C and transitioned to 28°C on day 2. Rhythm power was defined as the amplitude of the periodogram line at the dominant period minus the χ^2^ significance line at a significance of p < 0.01.

The same locomotor activity data collected for rest:activity rhythm determination were used to assess mean daily sleep and activity. Sleep was defined as 5 consecutive min of inactivity and sleep analysis was performed with PySolo software [68]. For each fly, mean total daily sleep amount was determined for 5 consecutive days (days 1–5 of DD for flies entrained at 25°C or days 2–6 of DD for flies entrained at 18°C), and then these individual means were averaged across all flies in a given group. Daily activity was determined by the mean number of DAM infrared beam breaks/min over the course of the same 5 days.

### Feeding:fasting rhythm analysis

To assess feeding:fasting rhythms, flies were reared and entrained as described for rest:activity rhythm analysis. Analysis of feeding:fasting rhythms was performed using the Fly Liquid-food Interaction Counter (FLIC) [24]. Liquid food used in the experiments was a 10% sucrose solution with 45 mg/L MgCl_2_ to provide additional ions for a more robust feeding signal. FLIC monitors were modified to be suitable for the duration of a multi-day experiment by attaching a liquid food reservoir (50 mL cell culture vial, CELLSTAR) to the base plate of each FLIC monitor. Flies were mouth-aspirated into FLIC monitor chambers with fly locations randomized by genotype. An equal number of flies per genotype were aspirated into each FLIC monitor. FLIC monitors were loaded into a temperature- and humidity-controlled incubator (Shel Labs) and exposed to 1 day of LD conditions to allow the flies to acclimate. Following acclimation, flies were exposed to DD conditions for 7 days. Two types of FLIC monitors were used to record feeding behavior: FLICv2.1 produced by the Pletcher lab at University of Michigan (used in Figs 1, 3, 4A–4D, 7, 8A and 8B), and Sable FLIC, produced by Sable Systems International (used in Figs 4G–4H, 6 and 8C). The two monitor types have slight differences in data processing due to the different electronic components used in constructing each type. Feeding events were defined based on two criteria for each FLIC monitor type: 1) amplitude readings exceeded the baseline by a set threshold (40 mV for FLICv2.1, 5 mV for Sable FLIC) for a minimum of 1 second, and 2) at some point during the event, amplitude readings achieved a minimum feeding threshold above baseline (85 mV for FLICv2.1, 15 mV for Sable FLIC). The duration of each feeding event was defined as the amount of time in which the amplitude reading remained above the set threshold. The number of feeding events was binned into 30-min intervals for analysis. Feeding:fasting rhythms for individual flies were calculated using χ^2^ periodogram analysis for days 1–6 of DD (experimental days 2–7) for flies entrained at 25°C or days 2–7 of DD (experimental days 3–8) for flies entrained at 18°C and transitioned to 28°C on day 2. Note that rhythm power as determined by χ^2^ periodogram is a relative value that is highly sensitive to bin length. Thus, power values for rest:activity analysis, for which we used a bin length of 1 min, are much higher than those obtained for feeding:fasting analysis, for which we used a 30-min bin length. Power values also systemically differed for data obtained by FLICv2.1 and Sable FLIC monitors, with Sable FLIC data producing higher power rhythms, even for flies of the same genotype. To facilitate comparisons between flies run in different monitor types we therefore normalized feeding:fasting rhythm power by dividing each fly’s rhythm power by the mean power of genetic control flies run in the same experiment. If multiple control lines were used in a given experiment, the normalization factor was the mean power value across all control flies. Flies were determined to be rhythmic (raw power > 10 for FLICv2.1, > 25 for Sable FLIC) or arrhythmic (raw power < 10 for FLICv2.1, < 25 for Sable FLIC) for each monitor type. Rhythmicity standards were determined empirically based on rhythm power observed in *cycle* mutant flies with no functional molecular clock. We also determined the total feeding duration by summing the time each fly spent in contact with food for the entirety of the experiment as a proxy for the total amount of food consumed per fly [24].

### Capillary Feeding (CAFE) assay

Feeding volume was recorded using CAFE assay as previously described [34]. Flies were reared and entrained as detailed above. Following entrainment, groups of 5–6 adult male flies, 5–10 days old, were housed in a humidified vial (*Drosophila* narrow vial, VWR International) containing a single calibrated glass micropipette (5 μL, VWR International) suspended in a hole at the top of the vial. The micropipette was filled by capillary action with the same liquid food as used in the FLIC assays (10% sucrose with 45 mg/L MgCl_2_ plus blue dye for ease of visualization). Flies were mouth-aspirated into a CAFE vial during the light period of their entrainment cycle and then transferred to DD conditions in a humidity- and temperature-controlled incubator. Flies were allowed a 24-hr acclimation period and then feeding data was recorded for the second 24 hr DD cycle. Capillary tubes were replaced every 24 hrs for experiments conducted at 25°C or every 12 hrs for experiments conducted at 28°C, to accommodate for additional evaporative loss. Loss of liquid food via evaporation was controlled by subtracting measurements from identical CAFE vials with no flies present. Average per fly liquid food consumption was determined based on measurements of the starting and ending meniscus of the food level in the capillary tube divided by the number of flies alive at the end of the feeding period.

### Determination of fly weights

Male flies that had eclosed within 48 hours of each other were placed in vials of standard cornmeal:molasses food at 25°C and exposed to 12:12 LD conditions. Flies were then transferred to vials of 10% sucrose/2% agar food to match the diet of FLIC and CAFE experiments, and moved to DD conditions for 7 days. Weight measurements were performed on the seventh day of DD. Flies were snap frozen in liquid nitrogen, thawed, and weighed in groups of five on an XSE105DU analytical balance (Mettler Toledo). Weight readings were divided by five to attain mg/fly. Each sample represents data collected from a single five-fly group.

### Immunohistochemistry

Adult fly brains were dissected in cold phosphate-buffered saline with 0.1% Triton-X (PBST) and fixed in 4% formaldehyde for 20–35 min. Brains were rinsed 3 X 15 min with PBST, blocked for 60 min in 5% normal donkey serum in PBST (NDST), and incubated for 24 hrs at RT in primary antibody diluted in NDST. Brains were then rinsed 3 X 15 min in PBST, incubated for 24 hrs in secondary antibody diluted in NDST, rinsed 3 X 15 min in PBST, cleared for 5 min in 50% glycerol in PBST, and mounted in Vectashield. Primary antibodies were as follows: rabbit anti-GFP 1:1000 (Molecular Probes A-11122), rat anti-RFP 1:1000 (Chromotek 5F8), mouse anti-HA 1:250 (BioLegend 901501), rabbit anti-SIFa 1:4000 (gift of J. Veenstra), and guinea pig anti-PERIOD 1:1000 (UPR 1140; gift of A. Sehgal). Secondary antibodies were as follows: FITC donkey anti-rabbit 1:1000 (Jackson 711-095-152), Cy3 donkey anti-rat 1:1000 (Jackson 712-16-150), Cy5 donkey anti-mouse 1:1000 (Jackson 715-175-151) and Cy3 donkey anti-guinea pig 1:1000 (Jackson 706-165-148). Immunolabeled brains were visualized with a Fluoview 1000 confocal microscope (Olympus). Trans-Tango flies were raised at 18°C and dissected ~2 weeks post-eclosion to maximize signal intensity [25].

### Statistical analysis

All statistical tests were performed in R (3.6.0) [69]. Plots were generated using GraphPad Prism (8.2.1). 2–3 independent experiments were run for each behavioral analysis, and data from all flies of a given genotype that survived the duration of the experiment were pooled. DAM, FLIC, CAFE, and fly weight data were analyzed using a t-test (for experiments with only 2 groups run simultaneously) or a one-way analysis of variance (ANOVA) followed by Tukey’s multiple comparisons test (for experiments with 3 or more groups), and p < 0.05 was considered significant. All representative feeding or activity records were selected to reflect the mean rhythm strength of a given genotype. Thus, we chose individual records that displayed a rhythm power that fell within the 95% confidence interval of the means listed in S1–S4 Tables. We have included raw data files for DAM, FLIC and food consumption experiments in S1 and S2 datasets.

## Supporting information

S1 Fig SIFa+ cell activation is necessary to affect feeding:fasting rhythm strength. (A) Flies expressing the temperature sensitive dTrpA1 cation channel but maintained below the temperature threshold for dTrpA1 activation had no difference in feeding rhythms as compared to both genetic controls. (B) Representative single-fly feeding records are shown for experimental days 3–8 for the indicated genotypes. Flies were transferred to DD conditions and maintained at 21°C at the start of experimental day 2 for the duration of the experiment. Feeding records show number of feeding events in 30 min bins, and data are double plotted, with each line representing two days of data. Gray and black bars represent subjective day and night, respectively.
(TIF)

S2 Fig SIFa+ cells persist in *SIFa* mutant flies. (A-B) Representative maximum projection confocal images of the brain of an *SIFa*^*1*^ mutant fly with SIF+ cells labeled using *SIFa*-LexA>mcherry. (A-B) SIFa cell number and morphology are normal in *SIFa* mutants, indicated by staining for the mcherry protein (A; red), despite a lack of SIFa peptide, as determined by SIFa antibody (B; green). (C-D) Close-up image of the PI region of the brain from (A) with four mcherry+ cell bodies indicated (arrowheads). Note that the SIFa-LexA line has non-specific expression in cells in the brain in addition to the SIFa+ PI cells.
(TIF)

S3 Fig RNAi-mediated SIFa knockdown increases feeding duration. Total time in contact with liquid food in FLIC monitors over the course of a 6-d experiment is plotted for the indicated genotypes. One of the two SIFa RNAi lines (*SIFa* RNAi^1^) spent significantly more compared to both genetic controls. The second SIFa RNAi line (*SIFa* RNAi^2^) spent significantly more time in contact with the liquid food compared to one of two genetic controls. Dots represent individual fly data and lines are means ± 95% confidence interval. **<0.01, ****<0.0001, n.s. = non-significant, Tukey’s multiple comparisons test.
(TIF)

S4 Fig SIFa mutant flies are not hyperactive. (A-D) Activity index (mean beam breaks/min during wake time) is plotted for the indicated genotypes. (A) Activity index is unchanged in *DILP2>dTrpA1* flies compared to genetic controls. (B) *SIFa>dTrpA1* flies have significantly decreased activity index compared to genetic controls. (C-D) Activity index is unchanged in *SIFa* mutants and rescue flies compared to heterozygous controls. For all graphs, dots represent individual fly data and lines are means ± 95% confidence interval. ****<0.0001, Tukey’s multiple comparisons test.
(TIF)

S1 Table Effect of activation and silencing of DILP+ and SIFa+ cells and ablation of SIFa+ cells on feeding:fasting rhythms. Genotype, number of flies analyzed (N), % arrhythmic, mean feeding rhythm period and normalized power (± 95% confidence interval (CI)), and results of ANOVA with Tukey’s multiple comparisons test for rhythm power are listed. To simplify nomenclature, we have omitted the terms *GAL4* and UAS from some genotypes, and used the symbol “>” to indicate that a GAL4 (listed to the left of the “>”) is driving the expression of the transgene listed to the right of the “>”. As only rhythmic flies are included in mean period determination, n for these values are listed in parenthesis in cases where it differs from the total n for the genotype. For statistical testing, *p* values reaching significance (<0.05) are bolded and the experimental genotype is in red font.
(DOCX)

S2 Table Effect of activation and silencing of DILP+ and SIFa+ cells and ablation of SIF+ cells on rest:activity rhythms. Genotype, number of flies analyzed (N), % arrhythmic, mean rest:activity rhythm period and power (± 95% confidence interval (CI)), and results of ANOVA with Tukey’s multiple comparisons test for rhythm power are listed. To simplify nomenclature, we have omitted the terms *GAL4* and UAS from some genotypes, and used the symbol “>” to indicate that a GAL4 (listed to the left of the “>”) is driving the expression of the transgene listed to the right of the “>”. As only rhythmic flies are included in mean period determination, n for these values are listed in parenthesis in cases where it differs from the total n for the genotype. For statistical testing, *p* values reaching significance (<0.05) are bolded and the experimental genotype is in red font.
(DOCX)

S3 Table Effect of *SIFa* mutations and RNAi-mediated knockdown on feeding:fasting rhythms. Genotype, number of flies analyzed (N), % arrhythmic, mean feeding rhythm period and normalized power (± 95% confidence interval (CI)), and results of T-test or ANOVA with Tukey’s multiple comparisons test for rhythm power are listed. To simplify nomenclature, we have omitted the terms *GAL4* and UAS from some genotypes, and used the symbol “>” to indicate that a GAL4 (listed to the left of the “>”) is driving the expression of the transgene listed to the right of the “>”. As only rhythmic flies are included in mean period determination, n for these values are listed in parenthesis in cases where it differs from the total n for the genotype. For statistical testing, *p* values reaching significance (<0.05) are bolded and the experimental genotype is in red font.
(DOCX)

S4 Table Effect of *SIFa* mutations and RNAi-mediated knockdown on rest:activity rhythms. Genotype, number of flies analyzed (N), % arrhythmic, mean rest:activity rhythm period and power (± 95% confidence interval (CI)), and results of T-test or ANOVA with Tukey’s multiple comparisons test for rhythm power are listed. To simplify nomenclature, we have omitted the terms *GAL4* and UAS from some genotypes, and used the symbol “>” to indicate that a GAL4 (listed to the left of the “>”) is driving the expression of the transgene listed to the right of the “>”. As only rhythmic flies are included in mean period determination, n for these values are listed in parenthesis in cases where it differs from the total n for the genotype. For statistical testing, *p* values reaching significance (<0.05) are bolded and the experimental genotype is in red font.
(DOCX)

S1 Dataset Raw data for DAM and FLIC behavioral experiments. (XLSX)

S2 Dataset Raw data for feeding duration and food consumption experiments. (XLSX)
