# Supplementary figures and images for: A circadian output center controlling feeding:fasting rhythms in Drosophila
Source: PLoS Genet. 2019 Nov 6;15(11):e1008478. doi: 10.1371/journal.pgen.1008478 (PMC6860455; doi:10.1371/journal.pgen.1008478)

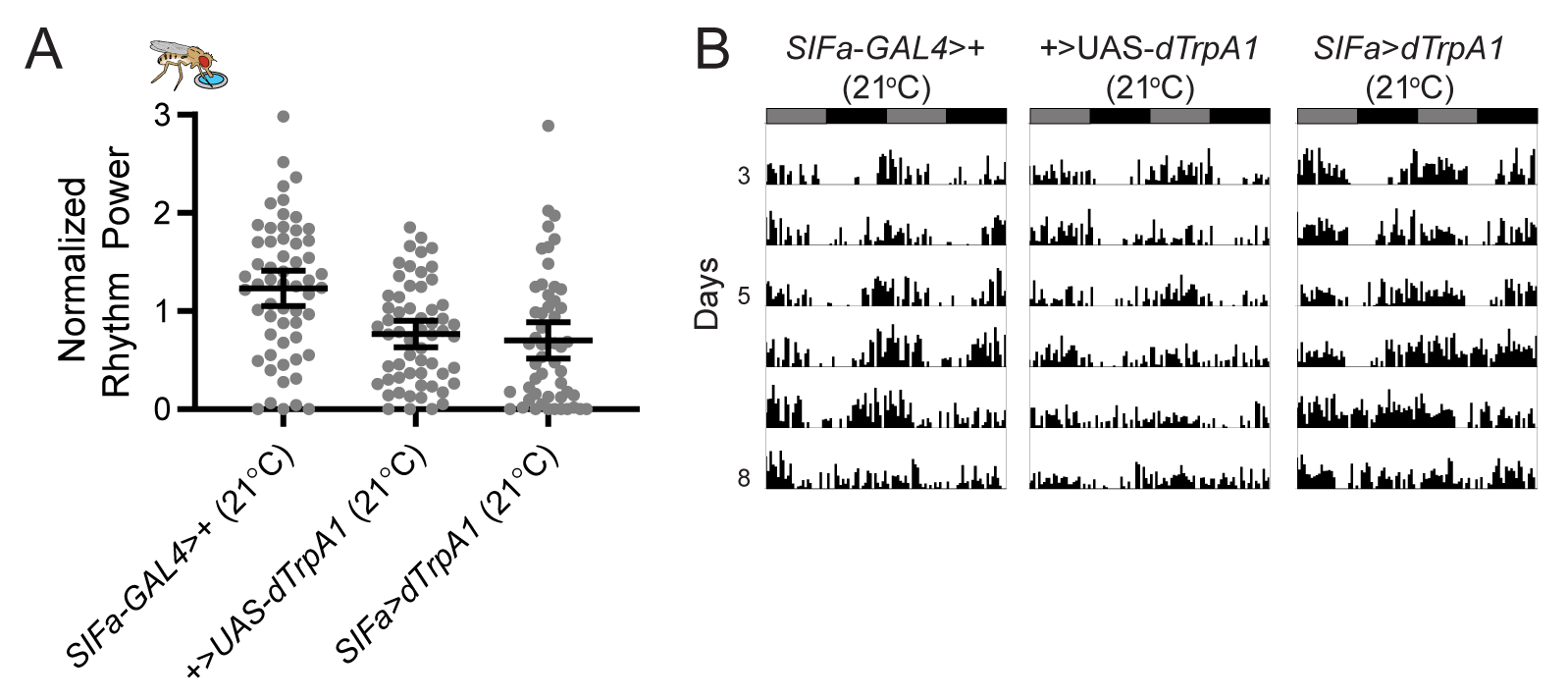

Supplement: S1 Fig — (A) Flies expressing the temperature sensitive dTrpA1 cation channel but maintained below the temperature threshold for dTrpA1 activation had no difference in feeding rhythms as compared to both genetic controls. (B) Representative single-fly feeding records are shown for experimental days 3–8 for the indicated genotypes. Flies were transferred to DD conditions and maintained at 21°C at the start of experimental day 2 for the duration of the experiment. Feeding records show number of feeding events in 30 min bins, and data are double plotted, with each line representing two days of data. Gray and black bars represent subjective day and night, respectively. (TIF) [file pgen.1008478.s001.tif]

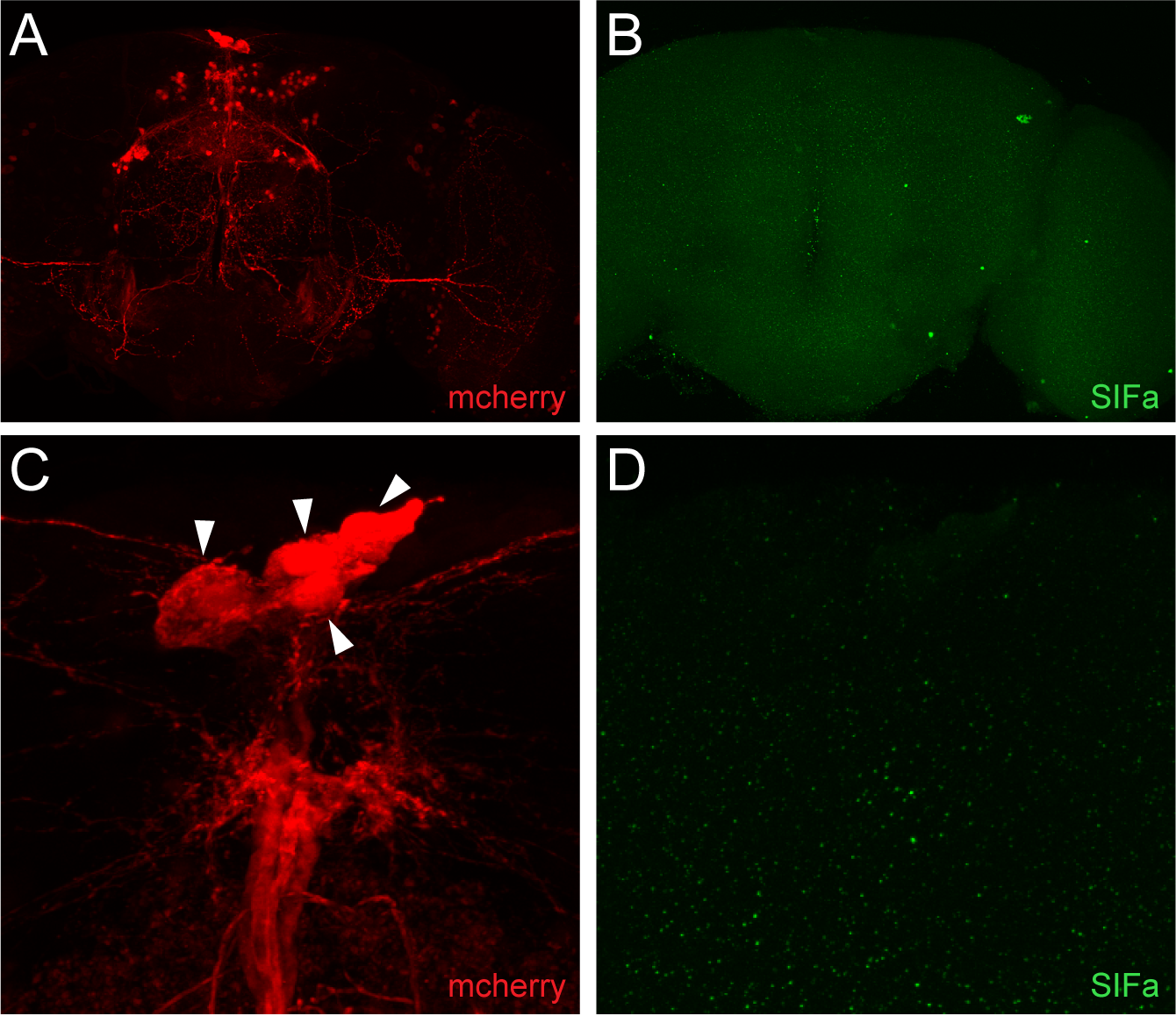

Supplement: S2 Fig — (A-B) Representative maximum projection confocal images of the brain of an SIFa1 mutant fly with SIF+ cells labeled using SIFa-LexA>mcherry. (A-B) SIFa cell number and morphology are normal in SIFa mutants, indicated by staining for the mcherry protein (A; red), despite a lack of SIFa peptide, as determined by SIFa antibody (B; green). (C-D) Close-up image of the PI region of the brain from (A) with four mcherry+ cell bodies indicated (arrowheads). Note that the SIFa-LexA line has non-specific expression in cells in the brain in addition to the SIFa+ PI cells. (TIF) [file pgen.1008478.s002.tif]

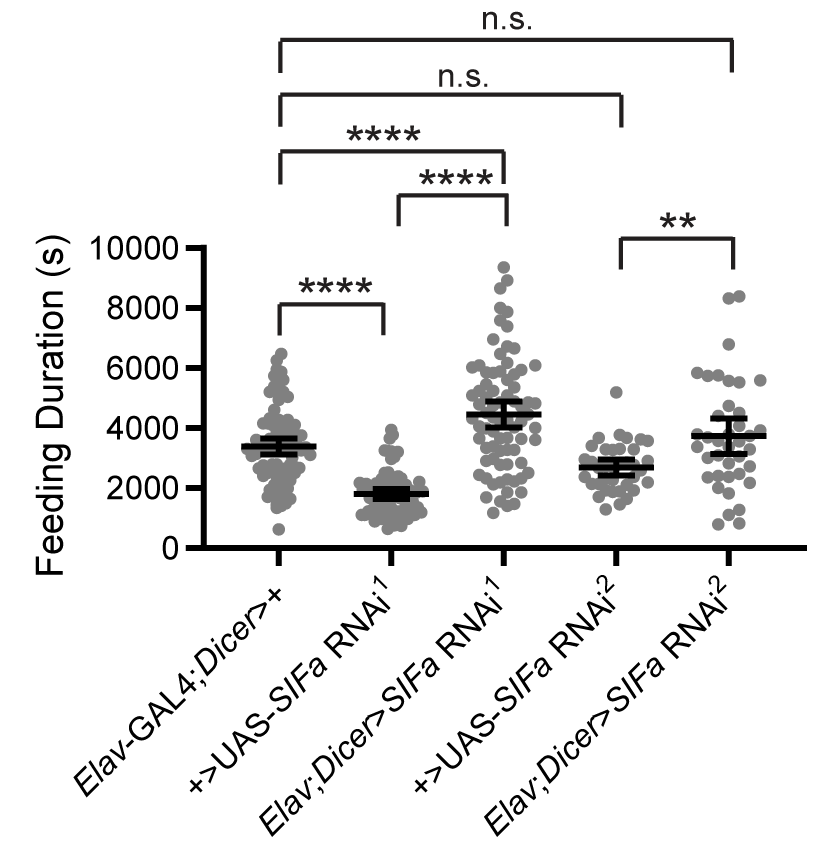

Supplement: S3 Fig — Total time in contact with liquid food in FLIC monitors over the course of a 6-d experiment is plotted for the indicated genotypes. One of the two SIFa RNAi lines (SIFa RNAi1) spent significantly more compared to both genetic controls. The second SIFa RNAi line (SIFa RNAi2) spent significantly more time in contact with the liquid food compared to one of two genetic controls. Dots represent individual fly data and lines are means ± 95% confidence interval. **<0.01, ****<0.0001, n.s. = non-significant, Tukey’s multiple comparisons test. (TIF) [file pgen.1008478.s003.tif]

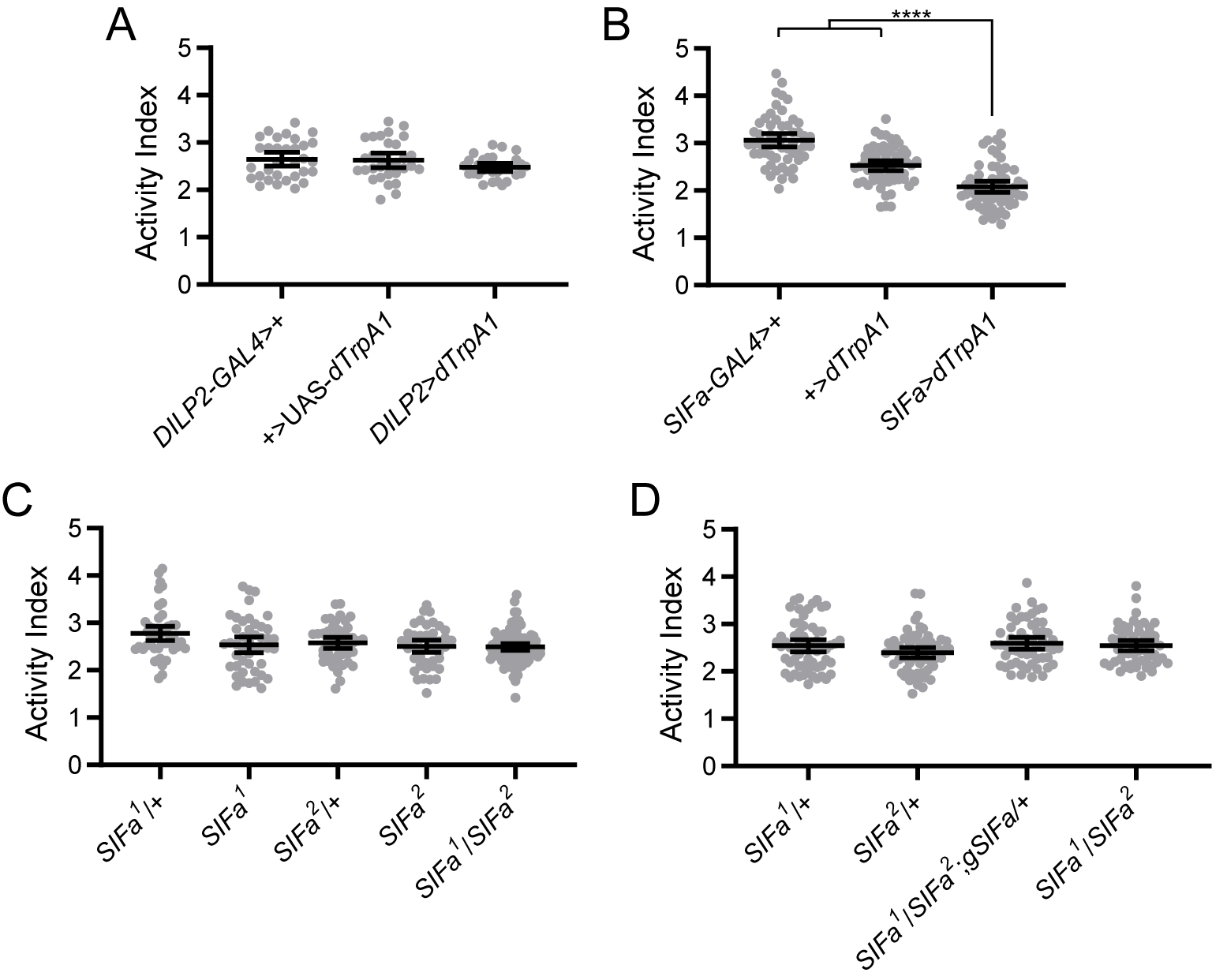

Supplement: S4 Fig — (A-D) Activity index (mean beam breaks/min during wake time) is plotted for the indicated genotypes. (A) Activity index is unchanged in DILP2>dTrpA1 flies compared to genetic controls. (B) SIFa>dTrpA1 flies have significantly decreased activity index compared to genetic controls. (C-D) Activity index is unchanged in SIFa mutants and rescue flies compared to heterozygous controls. For all graphs, dots represent individual fly data and lines are means ± 95% confidence interval. ****<0.0001, Tukey’s multiple comparisons test. (TIF) [file pgen.1008478.s004.tif]
