# Supplementary material for: A circadian output center controlling feeding:fasting rhythms in Drosophila
Source: PLoS Genet. 2019 Nov 6;15(11):e1008478. doi: 10.1371/journal.pgen.1008478 (PMC6860455; doi:10.1371/journal.pgen.1008478)
Supplement: S1 Table — Genotype, number of flies analyzed (N), % arrhythmic, mean feeding rhythm period and normalized power (± 95% confidence interval (CI)), and results of ANOVA with Tukey’s multiple comparisons test for rhythm power are listed. To simplify nomenclature, we have omitted the terms GAL4 and UAS from some genotypes, and used the symbol “>” to indicate that a GAL4 (listed to the left of the “>”) is driving the expression of the transgene listed to the right of the “>”. As only rhythmic flies are included in mean period determination, n for these values are listed in parenthesis in cases where it differs from the total n for the genotype. For statistical testing, p values reaching significance (<0.05) are bolded and the experimental genotype is in red font. (DOCX) [file pgen.1008478.s005.docx]

| Supplementary Table 1. Effect of activation and silencing of DILP+ and SIFa+ cells and ablation of SIFa+ cells on feeding:fasting rhythms | | | | | |
| --- | --- | --- | --- | --- | --- |
| Feeding:Fasting Rhythms - Activation | | |  | | |
| Genotype | N | % Arrhythmic | Period ± 95% CI | Normalized Power  ± 95% CI | Normalized Power ANOVA and Tukey HSD Tests |
| *DILP2-GAL4*>+ | 54 | 0 | 23.47 ± 0.20 | 1.10 ± 0.10 | ANOVA: F_(2,157)_= 4.57, *p* = **0.0117**  Tukey HSD *p*-values:  DILP2>+ : DILP2>dTrpA1 = 0.987  +>dTrpA1 : DILP2>dTrpA1 = **0.0238**  DILP2>+ : +>dTrpA1 = **0.0313** |
| *DILP2*>*dTrpA1* | 50 | 0 | 23.25 ± 0.08 | 1.11 ± 0.12 |  |
| +>UAS-*dTrpA1* | 56 | 3.57 | 23.28 ± 0.10 (n=54) | 0.906 ± 0.11 |  |
| *SIF-GAL4*>+ | 55 | 3.64 | 23.44 ± 0.06 (n=53) | 1.04 ± 0.11 | ANOVA: F_(2,133)_=23.29, p = **<0.0001**  Tukey HSD *p*-values:  SIFa>+ : SIFa>dTrpA1 = **<0.0001**  +>dTrpA1 : SIFa>dTrpA1 = **<0.0001**  SIFa>+ : +>dTrpA1 =0.5843 |
| *SIF*>*dTrpA1* | 37 | 35.14 | 23.13 ± 0.24 (n=24) | 0.470 ± 0.15 |  |
| *+*>UAS-*dTrpA1* | 44 | 0 | 23.38 ± 0.09 | 0.955 ± 0.11 |  |
| Feeding:Fasting Rhythms – Silencing | | |  |  |  |
| *DILP2-GAL4*>+ | 56 | 1.79 | 23.42 ± 0.07 (n=55) | 1.00 ±0.10 | ANOVA: F_(2,158)_=0.012, *p* = 0.988  Tukey HSD *p*-values:  DILP2>+ : DILP2>*Kir2.1*^TS^ = 0.988  +>*Kir2.1*^TS^ : DILP2>*Kir2.1*^TS^ = 0.999  DILP2>+ : +> *Kir2.1*^TS^ = 0.993 |
| *DILP2*>*Kir2.1^TS^* | 52 | 0 | 23.54 ± 0.09 | 0.993 ± 0.10 |  |
| *+*>UAS-*Kir2.1*^TS^ | 53 | 0 | 23.53 ± 0.06 | 0.996 ± 0.10 |  |
| *SIF-GAL4*>+ | 54 | 0 | 23.40 ± 0.07 | 1.13 ± 0.10 | ANOVA: F_(2,118)_ = 4.98, *p* = **0.0084**  Tukey HSD *p*-values:  SIFa>+ : SIFa>*Kir2.1*^TS^ = 0.0927  +>*Kir2.1^TS^* : SIFa>*Kir2.1*^TS^ = 0.9695  SIFa>+ : +>*Kir2.1*^TS^ = **0.0123** |
| *SIF*>UAS-*Kir2.1*^TS^ | 14 | 28.57 | 23.40 ± 0.68 (n=10) | 0.838 ± 0.38 |  |
| +>UAS-*Kir2.1*^TS^ | 53 | 1.89 | 23.42 ± 0.05 (n=52) | 0.870 ± 0.12 |  |
| Feeding:Fasting Rhythms – SIFa+ Ablation | | | | | |
| *SIF-GAL4*>+ | 55 | 1.82 | 23.95 ± 0.07 (n=54) | 0.972 ± 0.10 | ANOVA: F_(2,165)_15.31, *p* = **<0.0001**  Tukey HSD *p*-values:  SIF>+ : SIF>*Reaper* = **<0.0001**  +>*Reaper* : SIF>*Reaper* = **<0.0001**  SIF>+ : +>*Reaper* = 0.7063 |
| SIF>UAS-*Reaper* | 57 | 12.18 | 23.93 ± 0.05 (n=50) | 0.700 ± 0.10 |  |
| *+*>UAS-*Reaper* | 56 | 1.79 | 24.00 ± 0.05 (n=55) | 1.03 ± 0.10 |  |
