# Supplementary material for: A circadian output center controlling feeding:fasting rhythms in Drosophila
Source: PLoS Genet. 2019 Nov 6;15(11):e1008478. doi: 10.1371/journal.pgen.1008478 (PMC6860455; doi:10.1371/journal.pgen.1008478)
Supplement: S4 Table — Genotype, number of flies analyzed (N), % arrhythmic, mean rest:activity rhythm period and power (± 95% confidence interval (CI)), and results of T-test or ANOVA with Tukey’s multiple comparisons test for rhythm power are listed. To simplify nomenclature, we have omitted the terms GAL4 and UAS from some genotypes, and used the symbol “>” to indicate that a GAL4 (listed to the left of the “>”) is driving the expression of the transgene listed to the right of the “>”. As only rhythmic flies are included in mean period determination, n for these values are listed in parenthesis in cases where it differs from the total n for the genotype. For statistical testing, p values reaching significance (<0.05) are bolded and the experimental genotype is in red font. (DOCX) [file pgen.1008478.s008.docx]

| Supplementary Table 4. Effect of *SIFa* mutations and RNAi-mediated knockdown on rest:activity rhythms | | | | | |
| --- | --- | --- | --- | --- | --- |
| Rest:Activity Rhythms – Mutants | | | | | |
| Genotype | N | % Arrhythmic | Period ± 95% CI | Power ± 95% CI | Power T-test |
| *SIFa^1^* | 45 | 11.11 | 23.94 ± 0.12 (n=40) | 577.0 ± 114.1 | T_(84.52)_ = -11.78, *p* = **<0.0001** |
| *SIFa^1^*/+ | 47 | 0 | 23.81 ± 0.04 | 1717.2 ± 151.6 |  |
| *SIFa^2^* | 46 | 2.17 | 23.66 ± 0.10 (n=45) | 703.6 ± 134.5 | T_(91.59)_ = -7.083, *p* = **<0.0001** |
| *SIFa^2^*/+ | 48 | 0 | 23.68 ± 0.06 | 1423.9 ± 147.1 |  |
| Rest:Activity Rhythms – Genetic Rescue | | | | | |
| Genotype | N | % Arrhythmic | Period ± 95% CI | Power ± 95% CI | Power ANOVA and Tukey HSD Tests |
| *SIFa^1^*/+ | 60 | 0 | 23.90 ± 0.05 | 1724.1 ± 146.2 | ANOVA: F_(3,230)_ = 41.05, *p* = **<0.0001**  Tukey HSD *p*-values:  SIFa^1^/+ : SIFa^2^/+ = **0.0393**  SIFa^1^/+ : *Rescue* = **0.0026**  SIFa^1^/+ : SIFa^1^/SIFa^2^ = **<0.0001**  SIFa^2^/+ : *Rescue* = **<0.0001**  SIFa^2^/+ : SIFa^1^/SIFa^2^ = **<0.0001**  SIFa^1^/SIFa^2^ : *Rescue* = **<0.0001** |
| *SIFa^2^*/+ | 61 | 0 | 23.83 ± 0.07 | 1446.9 ± 143.7 |  |
| *SIFa^2^*/*SIFa^1^;gSIFa*/+ | 56 | 0 | 23.89 ± 0.05 | 2099.1 ± 164.6 |  |
| *SIFa^1^*/*SIFa^2^* | 57 | 0 | 23.80 ± 0.10 | 949.8 ± 127.1 |  |
| Rest:Activity Rhythms – RNAi | | | | | |
| Genotype | N | % Arrhythmic | Period ± 95% CI | Power ± 95% CI | Power ANOVA and Tukey HSD Tests |
| *Elav-GAL4;*UAS-*Dicer* >+ | 59 | 0 | 23.53 ± 0.11 | 560.2 ± 76.0 | ANOVA: F_(4,254)_ = 37.11, *p* = **<0.0001**  Tukey HSD *p*-values:  *Elav;Dicer>+* : +>*SIFa* RNAi^1^ = **<0.0001**  *Elav;Dicer>+* : +>*SIFa* RNAi^2^ = **<0.0001**  *Elav;Dicer>+* : *Elav;Dicer* >*SIFa* RNAi^1^ = **<0.0001**  *Elav;Dicer>+* : *Elav;Dicer* >*SIFa* RNAi^2^ = **<0.0001**  *+>SIFa RNAi^1^* : *Elav;Dicer* >*SIFa RNAi^1^* = **<0.0001**  *+>SIFa* RNAi^2^ : *Elav;Dicer* >*SIFa* RNAi^2^ = **<0.0001** |
| *+>*UAS-*SIFa* RNAi^1^ | 59 | 0 | 23.4 ± 0.28 | 850.0± 112.7 |  |
| *Elav;Dicer >SIFa* RNAi^1^ | 56 | 19.64 | 23.5 ± 0.55 (n=46) | 236.5 ± 49.6 |  |
| *+>*UAS-*SIFa* RNAi*^2^* | 59 | 0 | 23.8 ± 0.08 | 698.7 ± 99.1 |  |
| *Elav;Dicer >SIFa RNAi^2^* | 25 | 44 | 24.3 ± 1.2 (n=14) | 155.1 ± 45.7 |  |
